# Skeletal light-scattering accelerates bleaching response in reef-building corals

**DOI:** 10.1186/s12898-016-0061-4

**Published:** 2016-03-21

**Authors:** Timothy D. Swain, Emily DuBois, Andrew Gomes, Valentina P. Stoyneva, Andrew J. Radosevich, Jillian Henss, Michelle E. Wagner, Justin Derbas, Hannah W. Grooms, Elizabeth M. Velazquez, Joshua Traub, Brian J. Kennedy, Arabela A. Grigorescu, Mark W. Westneat, Kevin Sanborn, Shoshana Levine, Mark Schick, George Parsons, Brendan C. Biggs, Jeremy D. Rogers, Vadim Backman, Luisa A. Marcelino

**Affiliations:** Department of Civil and Environmental Engineering, Northwestern University, 2145 Sheridan Road, Evanston, IL 60208 USA; Department of Zoology, Field Museum of Natural History, 1400 South Lake Shore Drive, Chicago, IL 60605 USA; Department of Biomedical Engineering, Northwestern University, 2145 Sheridan Road, Evanston, IL 60208 USA; Keck Biophysics Facility, Northwestern University, 633 Clark Street, Evanston, IL 60208 USA; Fishes Department, John G. Shedd Aquarium, 1200 South Lake Shore Drive, Chicago, IL 60605 USA; Division of Water Resource Management, Florida Department of Environmental Protection, 2600 Blair Stone Road, Tallahassee, 32399 USA

**Keywords:** Global climate change, Optical scattering, Coral bleaching, Photosynthesis, Symbiosis

## Abstract

**Background:**

At the forefront of ecosystems adversely affected by climate change, coral reefs are sensitive to anomalously high temperatures which disassociate (bleaching) photosynthetic symbionts (*Symbiodinium*) from coral hosts and cause increasingly frequent and severe mass mortality events. Susceptibility to bleaching and mortality is variable among corals, and is determined by unknown proportions of environmental history and the synergy of *Symbiodinium*- and coral-specific properties. *Symbiodinium* live within host tissues overlaying the coral skeleton, which increases light availability through multiple light-scattering, forming one of the most efficient biological collectors of solar radiation. Light-transport in the upper ~200 μm layer of corals skeletons (measured as ‘microscopic’ reduced-scattering coefficient, $\mu ^{\prime}_{{S,m}}$), has been identified as a determinant of excess light increase during bleaching and is therefore a potential determinant of the differential rate and severity of bleaching response among coral species.

**Results:**

Here we experimentally demonstrate (in ten coral species) that, under thermal stress alone or combined thermal and light stress, low-$\mu ^{\prime}_{{S,m}}$ corals bleach at higher rate and severity than high-$\mu ^{\prime}_{{S,m}}$ corals and the *Symbiodinium* associated with low-$\mu ^{\prime}_{{S,m}}$ corals experience twice the decrease in photochemical efficiency. We further modelled the light absorbed by *Symbiodinium* due to skeletal-scattering and show that the estimated skeleton-dependent light absorbed by *Symbiodinium* (per unit of photosynthetic pigment) and the temporal rate of increase in absorbed light during bleaching are several fold higher in low-$\mu ^{\prime}_{{S,m}}$ corals.

**Conclusions:**

While symbionts associated with low-$\mu ^{\prime}_{{S,m}}$ corals receive less total light from the skeleton, they experience a higher rate of light increase once bleaching is initiated and absorbing bodies are lost; further precipitating the bleaching response. Because microscopic skeletal light-scattering is a robust predictor of light-dependent bleaching among the corals assessed here, this work establishes $\mu ^{\prime}_{{S,m}}$ as one of the key determinants of differential bleaching response.

**Electronic supplementary material:**

The online version of this article (doi:10.1186/s12898-016-0061-4) contains supplementary material, which is available to authorized users.

## Background

At the forefront of ecosystems adversely affected by climate change, coral reefs are sensitive to anomalously high temperatures which disassociate (bleaching) photosynthetic symbionts (*Symbiodinium*) from coral hosts and cause increasingly frequent and severe mass mortality events [1–4]. Susceptibility to bleaching and mortality is variable among corals [2, 5–8], and is partially determined (at unknown proportions) by a combination of environmental history [9, 10] and the interaction of *Symbiodinium*- [2, 11–14] and coral-specific [8, 15–19] properties (reviewed in [20]).

As photosynthetic performance of *in hospite Symbiodinium* is often impaired during thermally-induced bleaching (e.g., [21–23]), the interaction of temperature and irradiance exacerbate the bleaching response (reviewed in [4, 20, 24, 25]). Corals under thermal stress experience greater damage to the *Symbiodinium* photosynthetic apparatus (chronic photoinhibition of PSII) and elevated bleaching response when exposed to supra-optimal solar irradiances, indicating that temperature reduces the light intensity threshold for photoinhibition [4, 21, 25, 26].

*Symbiodinium* live within host tissues overlaying the coral skeleton, which can significantly increase light availability to symbionts through multiple scattering [15–18, 27], and together with within-tissue scatter and dynamic light redistribution (due to tissue contraction and scattering or absorption by host fluorescent pigments) [19, 28] form one of the most efficient biological collectors of solar radiation [15, 29]. This increase in light-availability is dependent on density and absorption properties of symbiont and host pigments and on diffuse reflectance of light from coral skeleton (*R*_*S*_) and tissue, which is mainly reliant on light scattering and absorption in the skeleton and tissue as well as overall coral morphology [15, 17–19, 27–33]. Scattering in skeletons (characterized by the reduced scattering coefficient, bulk-$\mu^{\prime}_{S}$ or $\mu^{\prime}_{S}$: inverse of the distance a photon travels before randomization) is mainly due to light interaction with skeletal microstructures throughout the entire skeleton (from 50 to 200 nm CaCO_3_ nanograins to 1–5 μm fiber bundles; [34, 35]) and larger length-scale structures (hundreds of micron size septa to millimeter size corallites; [15, 27, 36]). Furthermore, scattering in the superficial layer of coral skeletons (measured as microscopic-$\mu^{\prime}_{S}$ or $\mu ^{\prime}_{{S,m}}$: the inverse distance a short-path length photon travels before randomization [18, 37]) governs light-transport at sub-diffusion path lengths (~100 μm) and is affected by skeletal microstructures, but not larger length-scale structures [18]. Thus $\mu ^{\prime}_{{S,m}}$ can be described as $\mu^{\prime}_{S}$ of the skeletal material itself, within the top ~100 µm of the skeleton without voids [18]. Although *R*_*S*_ includes the effect of $\mu ^{\prime}_{{S,m}}$, it is primarily determined by $\mu^{\prime}_{S}$, absorption, and overall coral morphology [15, 18, 27, 29–31].

Greater total skeletal reflectance, associated with higher $\mu^{\prime}_{S}$, has been demonstrated to increase light-absorption by at least six times for symbionts *in hospite* and in simulations compared to those in vitro [15, 17]. By estimating absorption efficiency in differentially bleached corals and skeletal models (e.g., polished-laminae), it has been shown that skeletal light amplification (excess light available to the symbiont) is inversely related to symbiont concentration, leading to the prediction that skeletal $\mu^{\prime}_{S}$ could exacerbate the feedback of increasing photodamage for remaining *Symbiodinium* as symbiont densities diminish during bleaching (positive feedback-loop hypothesis) [15, 17, 29]. However, the rate of excess light increase as symbiont densities decrease has been demonstrated in models to be highly variable among corals, with high rates of excess light increase inversely correlated with skeletal $\mu ^{\prime}_{{S,m}}$ [18]. Low skeletal $\mu ^{\prime}_{{S,m}}$ values were significantly correlated with heightened bleaching susceptibility in a retrospective analysis of global bleaching events for 94 coral taxa, leading to the prediction that $\mu ^{\prime}_{{S,m}}$ (as the optical property responsible for the rate of feedback) is a potential determinant of the severity of bleaching response for this mechanism [18]. In this previous study, neither $\mu^{\prime}_{S}$ nor *R*_*S*_ were correlated with historical bleaching response [18].

To consolidate previous findings and provide predictions about the bleaching process that can be experimentally assessed, we propose the optical feedback hypothesis based on the effect of short-path light-transport. Although skeletal contribution to the endosymbiotic light microenvironment is normally small [38], skeletal optical properties become increasingly important as symbionts are lost and the skeleton becomes more exposed to light [18]. As densities of light absorbers (*Symbiodinium* cells and/or their photosynthetic pigments) decrease during the bleaching response, the coral skeleton becomes progressively exposed to downwelling light and dynamically becomes an increasingly significant source of excess light to remaining symbionts, compounding stress on *Symbiodinium* and provoking a more rapid and severe bleaching response. This feedback loop may proceed at differential rates that are determined by the rate at which the skeleton increases excess light to symbionts, as *Symbiodinium* and pigment concentrations decline [18]. As the optical property that is predictive of the rate of excess light increase as a function of pigment density, $\mu ^{\prime}_{{S,m}}$ affects the rate of feedback and may therefore be a determinate of bleaching severity [18]. We therefore predict that, depending on skeletal $\mu ^{\prime}_{{S,m}}$, corals that are bleaching should be differentially exposed to stress, and low-$\mu ^{\prime}_{{S,m}}$ corals should experience: (1) increased rates and severities of bleaching response, with *Symbiodinium* remaining *in hospite* showing increased rates and severities of light stress, and (2) increased skeleton-dependent light absorption by remaining *Symbiodinium*. Furthermore, (3) skeletal $\mu ^{\prime}_{{S,m}}$ should be a good predictor of the light-dependent bleaching effect but a poor predictor of temperature-dependent bleaching. These predictions of the optical feedback hypothesis have not been experimentally demonstrated among corals with diverse skeletal optical properties ($\mu ^{\prime}_{{S,m}}$ and *Rs*); which due to the dynamic nature of feedback, must be assessed as corals undergo bleaching.

Here we describe a heat- and light-stress experiment that demonstrates the effect of skeletal $\mu ^{\prime}_{{S,m}}$ on bleaching response using ten coral species selected for diversity of bleaching susceptibilities, skeletal optical properties, and *Symbiodinium* thermotolerances. By following the dynamics of holobiont response to stress directly, and developing a novel empirical model of skeleton-dependent light-absorption for *in hospite Symbiodinium*, we assessed the general predictions for coral bleaching under the optical feedback mechanism detailed above. The combined experimental and empirical modeling substantiates the predictions of the optical feedback hypothesis by establishing a connection between the dynamics of skeletal light amplification, bleaching response, *in hospite Symbiodinium* light absorption, and photophysiology among a diverse group of corals.

## Results

### Skeletal and holobiont optical characteristics

**Fig. 1:**
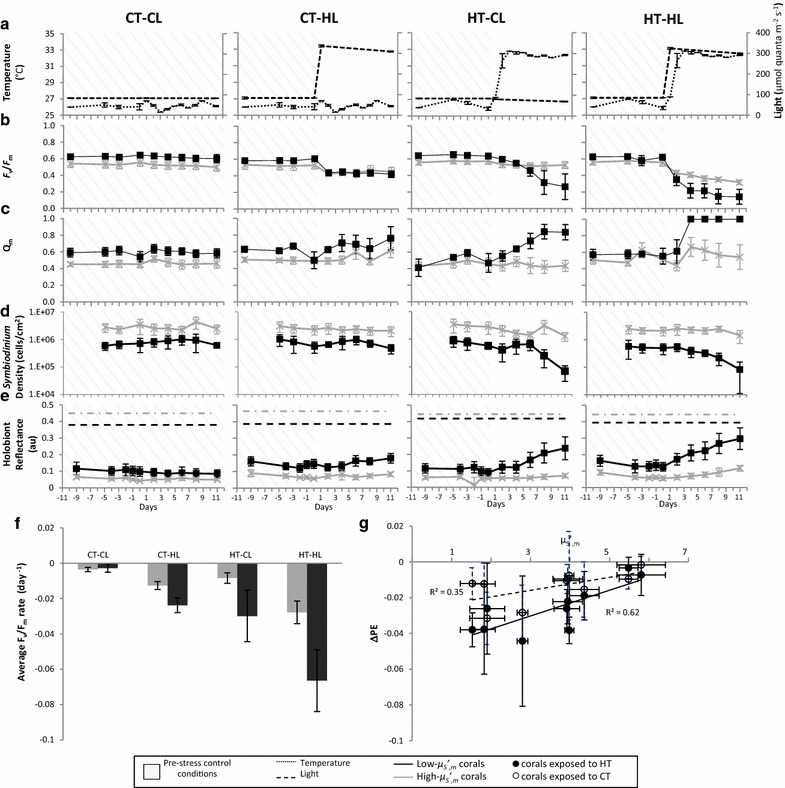
Dynamics of bleaching response variables. High- and low-$\mu ^{\prime}_{{S,m}}$ corals (means in *gray* and *black* respectively in **b**–**f**) responded differentially to experimental light (*broken line* in **a**) and temperature (*dotted line* in **a**) conditions (CT-CL: control temperature [26 °C] and light [83 μmol quanta m^−2^ s^−1^], CT-HL: control temperature and high light [328 μmol quanta m^−2^ s^−1^], HT-CL: high temperature [32 °C] and control light, and HT-HL: high temperature and high light; shaded areas are control). Under temperature stress (HT-CL and HT-HL), *Symbiodinium* *in hospite* of low-$\mu ^{\prime}_{{S,m}}$ corals experienced suppressed photosynthetic performance (**b**, **c**) and reduced cell density (**d**), and holobiont reflectance (**e**) of low-$\mu ^{\prime}_{{S,m}}$ corals approached the level of bare skeleton (*dashed lines* in **e** are post-experiment skeletal reflectance). Low-$\mu ^{\prime}_{{S,m}}$ corals experienced progressively greater average rates of photochemical efficiency loss (CT-CL *p* = 0.755, CT-HL *p* = 0.032, HT-CL *p* = 0.112, and HT-HL *p* = 0.042) as heat and light stress were combined (**f**). Isolating the effect of light from temperature on photochemical efficiency (**g**), $\mu ^{\prime}_{{S,m}}$ is correlated with the temporal rate of *F*_*v*_ */F*_*m*_ change $\left( {\varDelta PE \sim {{\varDelta^{ 2} \left( {F_{V} /F_{M} } \right)} \mathord{\left/ {\vphantom {{\varDelta^{ 2} \left( {F_{V} /F_{M} } \right)} {\left( {\varDelta t\varDelta I} \right)}}} \right. \kern-0pt} {\left( {\varDelta t\varDelta I} \right)}}} \right)$ expressed as the difference between CL and HL (Eq. 2) for corals exposed to HT (*filled circles*; *p* = 0.007) or CT (*open circles*; *p* = 0.07). All error bars are standard error of the mean

Microscopic scattering, $\mu ^{\prime}_{{S,m}}$, varied between 1.53 and 5.8 mm^−1^ (Table 1), with low-$\mu ^{\prime}_{{S,m}}$ corals (defined as below the mean of the ten species assessed: *Merulina* sp., *Pocillopora damicornis*, *Seriatopora hystrix*, and *Stylophora pistillata*) averaging 2.01 ± 0.27 mm^−1^ (mean ± std error) and high-$\mu ^{\prime}_{{S,m}}$ corals (*Diploria labyrinthiformis*, *Goniopora* sp., *Favia favus*, *Montipora foliosa*, and *Montipora digitata*) averaging 4.58 ± 0.34 mm^−1^. Consistent with the imperfectly-white coloration of the skeletons, *R*_*S*_ varied between 0.24 and 0.71 (relative to white standard, Table 1). Holobiont reflectance, *R*_*H*_, varied between 0.02 and 0.26 prior to the initiation of stress (Fig. 1a, e).

**Table 1:**
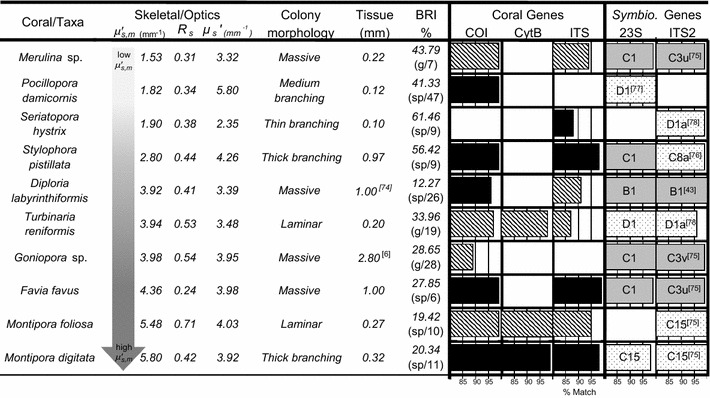
Optical, tissue, bleaching, and genetic data for individual corals

Skeletal optical properties [skeletal scattering ($\mu^{\prime}_{S,m}$), skeletal reflectance (*R*_*S*_)], and bulk scattering (*μ*_*S*_ʹ)], tissue thickness (all measured directly, except those annotated with citations [6, 74]), bleaching response index [BRI or the percent coral cover bleached and/or killed during mass bleaching events [18] used here as expected bleaching response for each taxon; parenthetical notation refers to genus- (g) or species-level (sp) estimations and the number of records that estimation is based upon], and genetic identity of corals and *Symbiodinium* assessed in experiment. Nucleotide sequences compared with Genbank (last accessed August 15, 2013) and reported as percent match (bar graphs) with accessions for coral mitochondrial cytochrome oxidase I (COI), cytochrome b (CytB), and nuclear internal transcribed spacer (ITS) genes; and *Symbiodinium* nuclear internal transcribed spacer region 2 (ITS2) and chloroplast 23S ribosomal (23S) genes. Shading of bars indicate the presence (solid black) or absence (diagonal lines) of the target species in Genbank, and low- (solid gray) or high-thermotolerance (stippled) of *Symbiodinium* [as reported in the literature (assuming C3u and C3v are similar to C3) [43, 75–78] and indicated by parenthetical superscript number on the phylotype used to categorize thermotolerance]

Although corals are highly complex structures, the variability detected in repeated measurements of $\mu ^{\prime}_{{S,m}}$, *R*_*S*_, and *R*_*H*_ is sufficiently small that we assume colonies can be characterized by mean values. The variability due to irregular surfaces and varying instrument positions is small, as is the coefficient of variation (COV), compared to the observed change in reflectance during bleaching. The average standard error of mean for *R*_*H*_ is <12 % (n = 10 measurements per ramet), and its COV is 38 % (standard deviation relative to mean) while the observed change in reflectance during bleaching increases as much as 300 % (Additional file 1: Figure S1a, f). This level of signal variability is sufficiently low to resolve changes in *R*_*H*_ as small as ~24 %. The COV of $\mu ^{\prime}_{{S,m}}$ for coral skeletons has been previously determined to be similarly small, at 12 % within a colony (assessing four areas from each of seven colonies) and 29.3 % within a species (assessing 4–8 colonies representing each of seven species) [18].

### Low $\mu^{\prime}_{S}$_, m_ corals experience increased rates and severities of bleaching and remaining *Symbiodinium* experience increased rates and severities of light stress

Corals in high temperature treatments (high temperature-control light: HT-CL, or high temperature-high light: HT-HL) experienced responses consistent with bleaching, with low-$\mu ^{\prime}_{{S,m}}$ corals bleaching at greater rates and severities. Under the application of temperature (HT-CL) or light and temperature (HT-HL) stress all corals experienced significant (ANOVA, *p* < 0.05) reductions in *Symbiodinium* cell densities (*ρ*) and increases in *R*_*H*_, with the most severe responses among low-$\mu ^{\prime}_{{S,m}}$ corals (Fig. 1; Additional file 2: Figure S2). Additionally, low-$\mu ^{\prime}_{{S,m}}$ corals experienced significantly (ANOVA, *p* < 0.001) greater decreases in *Symbiodinium* chlorophyll *a* densities (Chl *a*), with the greatest response occurring under the HT-HL treatment (Additional file 2: Figure S2). Exemplar *R*_*H*_ spectra over the visible (400–700 nm) and near infra-red (>700–800 nm) regions are shown in (Additional file 1) Figure S1 for *S. pistillata* (low-$\mu ^{\prime}_{{S,m}}$) and *M. digitata* (high-$\mu ^{\prime}_{{S,m}}$) before and after combined thermal- and light-stress was applied. As symbionts are lost during bleaching of *S. pistallata*, values of *R*_*H*_ approached the values of *R*_*S*_ (Fig. 1e; Additional file 2: Figure S1). Corals in the high light treatment alone (CT-HL) did not experience responses consistent with bleaching and observed differences in the dynamics of *R*_*H*_, *ρ*, or Chl *a* between low- and high-$\mu^{\prime}_{S}$_*, m*_ corals (Fig. 1; Additional file 2: Figure S2) are insignificant.

*Symbiodinium* that remained *in hospite* during bleaching experienced responses consistent with increasing light stress (i.e., corals under HT-CL, HT-HL), however *Symbiodinium* of low-$\mu^{\prime}_{S}$_*, m*_ corals experienced greater rates and severities of light stress (Fig. 1; Additional file 2: Figure S2). *Symbiodinium* associated with low-$\mu^{\prime}_{S}$_*, m*_ corals experienced significantly suppressed photochemical efficiency (*F*_*v*_*/F*_*m*_, linear mixed models, LMM, analysis) and elevated maximum-excitation pressure over PSII (*Q*_*m*_) (Fig. 1b, c, f). Specifically, the rate of reduction in photosynthetic performance [*Δ*(*F*_*V*_/*F*_*m*_)/*Δt* and *ΔQ*_*m*_/*Δt*] was significantly greater for *Symbiodinium* of low-$\mu ^{\prime}_{{S,m}}$ corals (clustered longitudinal analysis, $\mu ^{\prime}_{{S,m}}$-group × day interaction term *p* = 0.016 and 0.013, respectively: Fig. 1b, c; Table 2) and photosynthetic function diverged between low- and high-$\mu ^{\prime}_{{S,m}}$ corals at four and 2 days (for *F*_*v*_*/F*_*m*_ and *Q*_*m*_ respectively) after stress initiation (marginal analysis, *p* = 0.013 and 0.012, respectively, Fig. 1b, c; Tables 3). Although non-photochemical quenching (*Φ*_*NPQ*_) increased on average by 1.8-fold for low-$\mu ^{\prime}_{{S,m}}$ and 1.2-fold for high-$\mu ^{\prime}_{{S,m}}$ corals during bleaching, the dissipation of excess energy through non-photochemical mechanisms was not significantly different across high- and low-$\mu ^{\prime}_{{S,m}}$ corals (Additional file 2: Figure S2g).

**Table 2:** Hierarchical linear mixed models (LMM) analysis of photosynthetic performance

Metric of bleaching response	$\mu ^{\prime}_{{S,m}}$ Cluster	Rate (day^-1^)	*p* value, rate	CLA $\mu ^{\prime}_{{S,m}}$—day interaction term *p* value
*F*_*v*_ */F*_*m*_	Low-$\mu ^{\prime}_{{S,m}}$	−0.0319	<0.001	0.016
	High-$\mu ^{\prime}_{{S,m}}$	−0.0144	0.002	
*Q*_*m*_	Low-$\mu ^{\prime}_{{S,m}}$	0.043	<0.001	0.013
	High-$\mu ^{\prime}_{{S,m}}$	0.011	0.19	

Results of clustered longitudinal analysis (CLA) of high- and low-$\mu ^{\prime}_{{S,m}}$ corals. Marginal analysis of *F*_*v*_ */F*_*m*_ performed with values normalized to initial because the dynamic inversion of values (seen at day 4 in Fig. 1b; Additional file 3: Figure S3) makes marginal analysis insensitive to absolute differences over time

**Table 3:** Hierarchical linear mixed models (LMM) analysis of photosynthetic performance

Metric of bleaching response	Day after application of stress	Difference between high- and low-$\mu ^{\prime}_{{S,m}}$ groups	*p* value
*F*_*v*_/*F*_*m*_ (normalized to initial values)	0	0.0034	0.92
	2	0.054	0.074
	4	0.10	0.013
	6	0.15	0.011
*Q*_*m*_	0	−0.057	0.22
	2	−0.12	0.012
	4	−0.19	0.003
	6	−0.25	0.002

Results of marginal analysis of the photosynthetic performance (*F*_*v*_ */F*_*m*_ and *Q*_*m*_) of high- and low-$\mu ^{\prime}_{{S,m}}$ corals. Marginal analysis of *F*_*v*_ */F*_*m*_ performed with values normalized to initial because the dynamic inversion of values (seen at day 4 in Fig. 1b; Additional file 3: Figure S3) makes marginal analysis insensitive to absolute differences over time

### *Symbiodinium* of low $\mu^{\prime}_{S}$_, m_ corals experience increased rates of light absorption

We developed an empirical model of light absorption by *Symbiodiniumin hospite* by considering symbiont light-absorption (*I*_*a*_) as the sum of skeleton-independent absorption (*I*_*a*1_) of downwelling light and skeleton-dependent absorption (*I*_*a*2_) of reflected light (downwelling light not absorbed on the first pass and reflected by the skeleton back into coral tissue) [15–17]. The model relates *I*_*a*1_ and *I*_*a*2_ with parameters that were experimentally measured: skeletal reflectance, *R*_*S*_, of the clean skeleton and holobiont reflectance, *R*_*H*_, measured at different time points throughout the bleaching experiment.

**Fig. 2:**
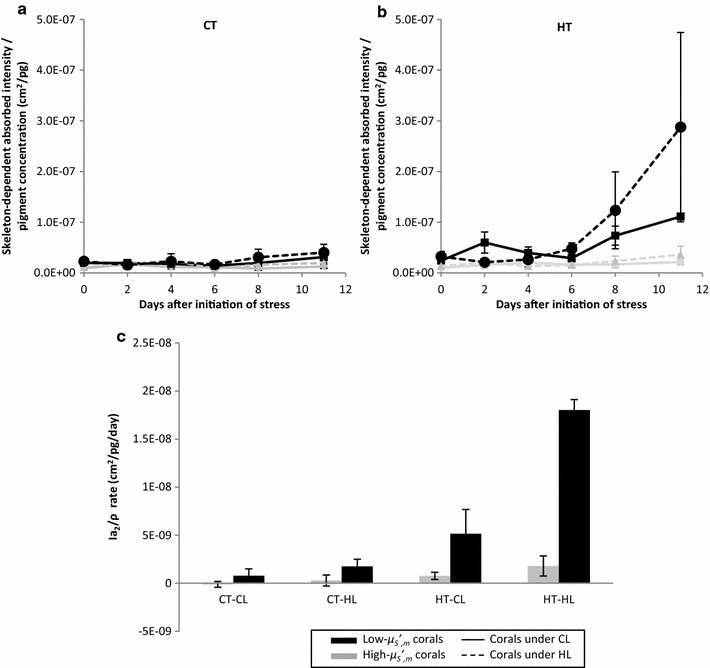
Dynamics of modeled *Symbiodinium* light absorption *in hospite* due to skeletal backscattering ($\mu ^{\prime}_{{S,m}}$). *Symbiodinium* *in hospite* of high- (*gray line*) and low-$\mu ^{\prime}_{{S,m}}$ (*black line*) corals are (conservatively) predicted by an empirical model to have differential skeleton-dependent light absorption per unit pigment (*I*_*a*2_/*ρ*). Under **a** CT, the absorption of light in high- and low-$\mu ^{\prime}_{{S,m}}$ corals is similar when exposed to CL (*solid line*) and HL (*broken line*). Under **b** HT, the absorption of light in low-$\mu ^{\prime}_{{S,m}}$ corals is several times larger under either light condition, but the increase under HL is dramatic. Additionally, the increase in (conservatively) estimated temporal rates of light absorbed per unit pigment $\left( {{{\varDelta \left( {{{I_{a2} } \mathord{\left/ {\vphantom {{I_{a2} } \rho }} \right.\kern-0pt} \rho }} \right)} \mathord{\left/ {\vphantom {{\varDelta \left( {{{I_{a2} } \mathord{\left/ {\vphantom {{I_{a2} } \rho }} \right. \kern-0pt} \rho }} \right)} {\varDelta t}}} \right. \kern-0pt}{\varDelta t}}} \right)$ in low-$\mu ^{\prime}_{{S,m}}$ corals (*black bars*) is progressively greater as heat and light stress were combined (**c**). All abbreviations follow Fig. 1 and error bars are standard error of the mean

The results of the model of *Symbiodinium* light absorption indicate that the estimated skeleton-dependent light absorbed per unit pigment (*I*_*a*2_/*ρ*) and its rate (Δ(*I*_*a*2_/*ρ*)/Δ*t*) were several fold higher in low-$\mu ^{\prime}_{{S,m}}$ corals (Fig. 2a–c, where average *ρ* for low- and high-$\mu ^{\prime}_{{S,m}}$ corals are concentrations of Chl *a* in μg/cm^2^, Additional file 2: Figure S2). This pattern remained (Fig. 2c) when the effect of downwelling light was isolated (subtracting *I*_*a*2_/*ρ* determined under CL from the HL treatment using Taylor expansion, Eq. 2 using *I*_*a*2_/*ρ* as a metric instead of change in photochemical efficiency). As symbiont densities decrease, *I*_*a*2_/*ρ* increases at a rate of −Δ(*I*_*a*2_/*ρ*|_*HTHL*_ − *I*_*a*2_/*ρ*|_*HTCL*_)/Δ*ρ*, which follows an inverse-power law function of $\mu ^{\prime}_{{S,m}}$ (r^2^ = 0.79), consistent with previously published data on flat-coral models [18]. Parameters chosen are valid at high per-cell pigment concentration, and *I*_*a*2_/*ρ* significantly underestimates actual values as *ρ* decreases. Because *ρ* is reduced in low-$\mu ^{\prime}_{{S,m}}$ corals during bleaching (Fig. 1d), our estimation of *I*_*a*2_ is conservative, and feedback effect is expected to be even more pronounced.

### Light and temperature dependent bleaching effects

The light- or temperature-dependent bleaching effects were evaluated for one parameter in particular; the rate of reduction in photochemical efficiency of *Symbiodinium* with bleaching (Δ*PE*). In the case of light-dependent bleaching effect, Δ*PE* for corals exposed to CL were subtracted from those exposed to HL for either control (i.e., CT-HL–CT-CL) or high (i.e., HT-HL–HT-CL) temperature (Eq. 2). Thereby, the effect of light on bleaching was determined by calculating the increased light stress [Δ*PE* (HL–CL)] in the absence and presence of thermal stress. The rate of light-induced reduction in photosynthetic efficiency Δ*PE* is positively correlated with $\mu ^{\prime}_{{S,m}}$, approaching 0 (no loss of *F*_*v*_*/F*_*m*_ with time) at the highest values of $\mu ^{\prime}_{{S,m}}$, under high (r^2^ = 0.62, *p* = 0.007) or control (r^2^ = 0.35, *p* = 0.07) temperature (Fig. 1g). Taking a similar approach to isolate the effect of temperature on the rate of reduction in photosynthetic efficiency, Δ*PE* of corals exposed to CT were subtracted from those exposed to HT for either control (i.e., HT-CL–CT-CL) or high (i.e., HT-HL–CT-HL) light (Eq. 3). Temperature-induced loss of *F*_*v*_*/F*_*m*_ over time, Δ*PE,* is not significantly correlated with $\mu ^{\prime}_{{S,m}}$ (r^2^ = 0.18, *p* = 0.23, Additional file 3: Figure S3a). Although all corals experienced some reduction in *F*_*v*_*/F*_*m*_ (during the 11 days of the experiment) under single stressor treatments (CT-HL and HT-CL), larger reductions were observed under combined heat and light stress with the greatest decline among low-$\mu ^{\prime}_{{S,m}}$ corals (Fig. 1b).

### Factors that did not influence bleaching response

The diversity of corals and symbionts included in these experiments permitted examination of the effects of several factors that have been previously described as determinants of bleaching response (*R*_*S*_, bulk-$\mu^{\prime}_{S}$, coral tissue thickness, colony morphology, *Symbiodinium* thermotolerance) and confounding factors of $\mu ^{\prime}_{{S,m}}$ (i.e., parameters that correlated with $\mu ^{\prime}_{{S,m}}$: *a priori* physiological differences observed among the targeted species during baseline pre-stress measurements, including *Symbiodinium* and Chl *a* densities, and photochemical efficiency). None of these factors were significantly correlated with the changes in photosynthetic performance observed in bleaching corals.

**Fig. 3:**
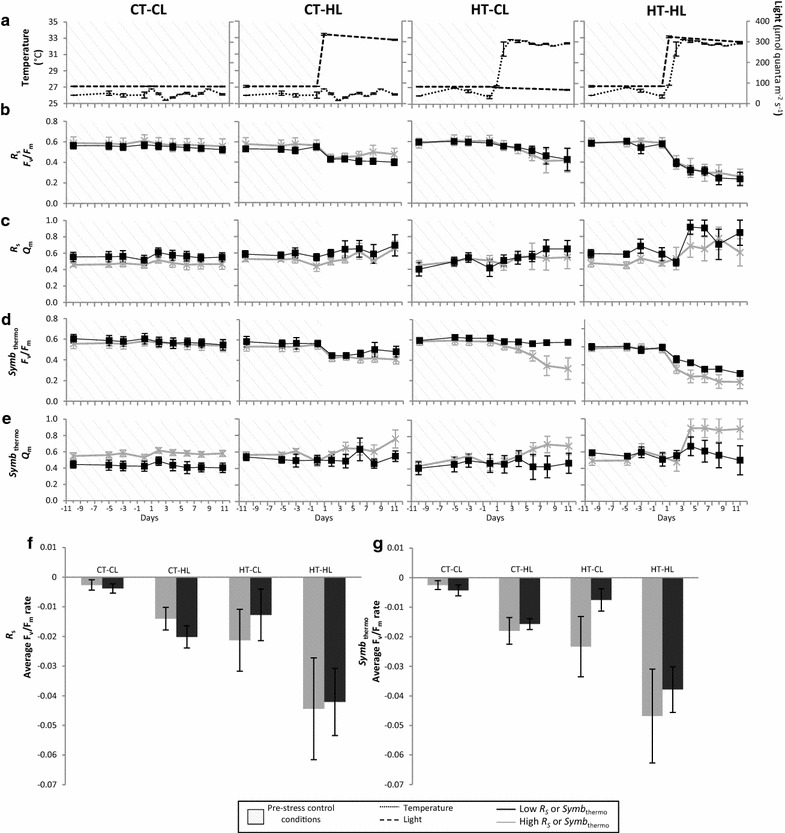
Effects of skeletal reflectance (*R*_*S*_) and *Symbiodinium* thermotolerance (*Symb*_thermo_) on photosynthetic performance dynamics. High- and low- (means in *gray* and *black* respectively in **b**–**g**) *R*_*S*_ and *Symb*_thermo_ corals responded similarly to experimental light (*broken line* in **a**) and temperature (*dotted line* in **a**) conditions (described in Fig. 1). Photosynthetic performance was similarly suppressed under increased stress in corals grouped by *R*_*S*_ (**b**, **c**) and was modestly (but non-significantly) more suppressed for corals hosting high-thermotolerance *Symbiodinium* (**d**, **e**). Both low- and high-*R*_*S*_ corals experienced a progressively greater average rate of photochemical efficiency loss (CLL *p* = 0.64; CHL *p* = 0.28; TLL *p* = 0.55 and THL *p* = 0.91) as heat and light stress were increased (**f**), and both low and high-*Symb*_thermo_ corals experienced a progressively greater average rate of photochemical efficiency loss (CLL *p* = 0.47; CHL *p* = 0.70; TLL *p* = 0.26 and THL *p* = 0.68) as heat and light stress were increased (**g**). All* error bars* are* standard error* of the mean

Corals examined included substantial diversity in *R*_*S*_, bulk-$\mu^{\prime}_{S}$, coral tissue thickness, colony morphology, and *Symbiodinium* thermotolerances (Table 1). Skeletal reflectance was not significantly associated with changes in *F*_*v*_*/F*_*m*_ or *Q*_*m*_ (Fig. 3b, c, f; Additional file 4: Figure S4; LMM, *p* > 0.15). Bulk-$\mu^{\prime}_{S}$ (Table 1) was not significantly associated with the rate of reduction in photosynthetic efficiency *ΔPE* (r^2^ = 0.02, p > 0.5). The experimental corals included thin (*S. hystrix*), medium (*P. damicornis*), and thick branching (*S. pistillata* and *M. digitata*) colony morphologies, as well as laminar (*M. foliosa* and *T. reniformis*) and massive (*Merulina* sp., *D. labyrinthiformis*, *Goniopora* sp., and *F. favus*) forms; however colony morphology was not significantly associated with light- (r^2^ = 0.001, *p* > 0.5) nor temperature- (r^2^ = 0.02, *p* > 0.5) dependent *ΔPE*. Coral tissue thickness varied between 0.1 and 2.8 mm (Table 1), but was not significantly associated with light- (r^2^ = 0.12, *p* > 0.5) nor temperature- (r^2^ = 0.05, *p* > 0.5) dependent *ΔPE*. Experimental corals hosted some of the highest (C8a, C15, D1 and D1a) or lowest (B1 and, assuming similar to C3, C3u and C3v) thermotolerance phylotypes known (Table 1). However *Symbiodinium* thermotolerance was not significantly associated with *F*_*v*_*/F*_*m*_ or *Q*_*m*_ (LMM, *p* > 0.05), and the observed trends have greater losses of photosynthetic performance among high-thermotolerance physiotypes (Fig. 3d, e, g; Additional file 5: Figure S5).

Physiological differences between low- and high-$\mu ^{\prime}_{{S,m}}$ corals were detected in the absence of stress: low-$\mu ^{\prime}_{{S,m}}$ corals had higher baselines for *F*_*v*_*/F*_*m*_ (Fig. 1b) and Chl *a* (Additional file 3: Figure S3c) and lower baselines for *Symbiodinium* density, *ρ*, (Fig. 1d) during monitoring prior to experimental manipulation (t test, all *p* < 0.001). Only baseline-*F*_*v*_*/F*_*m*_ had a significant correlation with *F*_*v*_*/F*_*m*_ (LMM, *p* = 0.01), and also correlated with *ΔPE* under HT (r^2^ = 0.45). However, this correlation was unstable and primarily caused by a single datapoint (*M. digitata*), without which r^2^ dropped to 0.12. Baseline-*F*_*v*_*/F*_*m*_ could not predict *ΔPE* under CT (r^2^ < 0.07), and the difference between mean *ΔPE* of baseline-low- and baseline-high-*F*_*v*_*/F*_*m*_ was not significant (*p* > 0.25 versus 0.007 for $\mu ^{\prime}_{{S,m}}$ as the explanatory variable).

## Discussion

Results of the bleaching experiment and empirical light-absorption model are consistent with predictions of the optical feedback hypothesis. Bleaching corals with skeletal nanostructures that scatter light at relatively low $\mu ^{\prime}_{{S,m}}$ experienced increased rates and severities of bleaching response (*ΔR*_*H*_, *ρ*, Chl *a*; Fig. 1d, e; Additional files 1 and 2: Figures S1, S2), light stress on retained *Symbiodinium* (*ΔF*_*v*_*/F*_*m*_, *Q*_*m*_; Fig. 1), and amounts and rates of skeleton-dependent light absorption by remaining *Symbiodinium* [(*I*_*a*2_/*ρ*) and (Δ(*I*_*a*2_/*ρ*)/Δ*t*); Fig. 2] relative to corals with skeletal nanostructures that scatter light at relatively high $\mu ^{\prime}_{{S,m}}$.

### Low $\mu^{\prime}_{S}$_, m_ corals experience increased rates and severities of bleaching and remaining *Symbiodinium* experience increased rates and severities of light stress

Although all corals experienced some response to increased temperature, differentially increased bleaching was detected among low-$\mu ^{\prime}_{{S,m}}$ corals as early as day 2 (under HL-HT) and no later than day 6 (under CL-HT) after initiation of stress (Fig. 1; Additional file 2: Figure S2). Similarly, differentially decreased photosynthetic performance of retained *Symbiodinium* was nearly simultaneous with bleaching (within the sampling periods of the experimental design) and was detected among low-$\mu ^{\prime}_{{S,m}}$ corals as early as day 2 (under HL-HT) and no later than day 6 (under CL-HT) of the experiment (Fig. 1; Additional file 2: Figure S2).

Change in photosynthetic performance was evaluated by measuring changes in *F*_*v*_*/F*_*m*_ and *Q*_*m*_ of all corals before and after the application of stress. *F*_*v*_*/F*_*m*_ indicates the proportion of potentially active PSII reaction centers under dark-adapted conditions [39] and significant decreases in *F*_*v*_*/F*_*m*_ over time under light- and heat-stress have been measured in bleaching corals (e.g., [4, 24, 25]). *Q*_*m*_ [40, 41] is sensitive to effective quantum yield (*Φ*_*PSII*_) oscillations as a result of the induction of multiple photoprotective pathways that compete for energy dissipation when light absorption exceeds photochemistry and indicates the proportion of active (or open) PSII reaction centers under peak irradiance [40]. Values approximating 0 indicate light-limitation with most reaction centers open, ≈1 indicate photoinhibition with most reaction centers closed, and photoacclimation is indicated when *Q*_*m*_ remains unchanged during suppressed photochemical efficiency [11, 12, 40]. Differential rates of divergence of photosynthetic performance (at day 4 for *F*_*v*_*/F*_*m*_ and day 2 for *Q*_*m*_; Fig. 1) indicate that *Symbiodinium* associated with high-$\mu ^{\prime}_{{S,m}}$ corals were experiencing photoacclimation (*Q*_*m*_ remains unchanged while *F*_*v*_*/F*_*m*_ decreases modestly) while those associated with low-$\mu ^{\prime}_{{S,m}}$ corals were experiencing photoinhibition (*Q*_*m*_ approaches one, while *F*_*v*_*/F*_*m*_ decreases significantly); consistent with observations of bleaching corals [11, 40].

All corals dissipated excess energy through *Φ*_*NPQ*_ at similar levels (increase of 1.2-fold to 1.8-fold after thermal stress, Additional file 2: Figure S2g). This finding may seem unexpected as *Φ*_*NPQ*_ is mainly affected by photoprotective pathways (downregulation of PSII antenna pigments and the xanthophyll cycle) [42], and given the increased light stress experienced by low-$\mu ^{\prime}_{{S,m}}$ corals, a greater increase in *Φ*_*NPQ*_ would be expected compared to high-$\mu ^{\prime}_{{S,m}}$ corals. However, while suppressed *F*_*v*_*/F*_*m*_ and elevated *Q*_*m*_ are often associated with severe bleaching response (e.g., [11, 12]), there is no consensus [43–46] that variation in *NPQ* is indicative of resistance [47, 48] or sensitivity [44] to thermal stress and photoinhibition.

### *Symbiodinium* associated with low-$\mu ^{\prime}_{{S,m}}$ corals absorb light at higher rates and amounts

We developed an empirical model of light-absorption for *inhospite Symbiodinium* to test the assumption that the susceptibility of low-$\mu ^{\prime}_{{S,m}}$ corals is driven by a feedback-loop between absorber loss (decrease in *ρ*) and the rate of light amplification increase, which exposes remaining symbionts to rapidly increasing light. The rate of light amplification increase is modeled as: $- \frac{{d\left( {{{I_{a} } \mathord{\left/ {\vphantom {{I_{a} } {I_{a1} }}} \right. \kern-0pt} {I_{a1} }}} \right)}}{d\rho }$; where *I*_*a*_ is the fraction of incident light absorbed in tissue and *I*_*a*1_ is the fraction incident absorbed on its first pass through tissue. Change in the rate of light amplification increase is a consequence of a higher rate of light absorption per pigment due to skeletal reflectance, which is modeled as: $\frac{{d\left( {{{I_{a2} } \mathord{\left/ {\vphantom {{I_{a2} } \rho }} \right. \kern-0pt} \rho }} \right)}}{d\rho }$; where *I*_*a*2_ = *I*_*a*_ − *I*_*a*1_ is the fraction of incident light not absorbed on the first pass, scattered by the skeleton back into the tissue and subsequently absorbed.

The empirical model of light-absorption for *in hospite Symbiodinium* is a generalization of prior models [15, 17], however it differentiates between downwelling (skeleton-independent) and reflected (skeleton-dependent) light-absorption so that the effect of skeletal optical properties on light intensity experienced by symbionts is explicitly estimated and repeated passes of light between tissue and skeleton can be accounted for. The model expresses *I*_*a*_, *I*_*a*1_ and *I*_*a*2_ through experimentally determined values for *R*_*S*_, *R*_*H*_, and three model parameters describing light transport properties of the holobiont (*α*, *β*, *γ*; see “Methods”). Downwelling light that is not absorbed during the first pass can be returned to tissues by the skeleton, lost to absorption, or diffusely scattered out of the colony [17, 19, 27, 28] and may repeatedly pass between skeleton and tissue (i.e., aided by skeletal morphology; [19, 30]). Thus, *I*_*a*2_ may be the result of multiple passes of light through tissue caused by multiple reflections of the skeleton [15, 17]. For a flat coral model (no multiple passes through tissue), and neglecting absorption of light reflected by the skeleton in tissue, our model (Eqs. 5, 6 and 7) converges to the approximate solution used to estimate the absorption of light based on holobiont and skeletal reflectance values [15, 29, 49].

The estimated *Symbiodinium* light absorption indicates that the effect of $\mu ^{\prime}_{{S,m}}$ on light absorption by *Symbiodinium*, *I*_*a*_, is substantial. Skeleton-dependent light absorbed per unit pigment (*I*_*a*2_/*ρ*) and its rate (Δ(*I*_*a*2_/*ρ*)/Δ*t*) were several fold higher in low-$\mu ^{\prime}_{{S,m}}$ corals (Fig. 2a–c). This pattern was even more pronounced for combined light and temperature stress (Fig. 2b) and remained when the effect of downwelling light was isolated (Fig. 2c) (subtracting *I*_*a*2_/*ρ* determined under CL from the HL treatment). Parameters (*α*, *β*, *γ*) chosen are valid at high per-cell pigment concentration and (*I*_*a*2_/*ρ*) significantly underestimates actual values as *ρ* decreases. Because *ρ* is greatly reduced in low-$\mu ^{\prime}_{{S,m}}$ corals during bleaching compared to high-$\mu ^{\prime}_{{S,m}}$ (Fig. 1d), these calculations are expected to underestimate *I*_*a*2_, and the feedback effect is expected to be even more pronounced.

### $\mu ^{\prime}_{{S,m}}$ is a robust predictor of light-dependent bleaching, but not of temperature-dependent bleaching

By mathematically isolating the effect of light on bleaching from temperature and other confounding factors, including those unknown (light-dependent bleaching effect), we found that the rate of reduction in photochemical efficiency during bleaching (Δ*PE*) is associated with $\mu ^{\prime}_{{S,m}}$, indicating that $\mu ^{\prime}_{{S,m}}$ is one of the determinants of light-dependent bleaching severity. The rate of light-induced loss of *F*_*v*_*/F*_*m*_ is much more pronounced in low-$\mu ^{\prime}_{{S,m}}$ corals; high-$\mu ^{\prime}_{{S,m}}$ corals are nearly invariable under HT or CT conditions (*ΔPE* approached 0; Fig. 1f, g). While $\mu ^{\prime}_{{S,m}}$ was a robust predictor of light-dependent bleaching as it explained 62 % of the variance in Δ*PE* for HT (r^2^ = 0.62, *p* = 0.007, Fig. 1g), it was not a robust predictor of the temperature-dependent bleaching as $\mu ^{\prime}_{{S,m}}$ explained only 18 % of the variance in Δ*PE* for HL (r^2^ = 0.18, *p* = 0.23, Additional file 3: Figure S3a).

Heat and light stress have a compounding effect on bleaching response; differential sensitivity to light is amplified by temperature (Fig. 1f, g) as excess light generated by skeletal scattering may overwhelm photosystems impaired by thermal stress. Heat reduces the ability of *Symbiodinium* to utilize light in photosynthesis [4, 23, 25, 50] and can uncouple energy absorption from photochemistry [23, 50]; resulting in excess energy independent of light increase. Therefore, *Symbiodinium* may perceive heat stress as an increase in excitation pressure over photosystem II [23, 50] and experience an increase in excess light as a result of an increase in temperature. In the absence of increased temperature stress, the effect of light-transport in the surface of the coral skeleton seems low, but once temperature increases and bleaching is initiated, the effect of light stress becomes remarkable, in particular for low-$\mu ^{\prime}_{{S,m}}$ corals (r^2^ for *ΔPE(*$\mu ^{\prime}_{{S,m}}$) is two times lower for CT than HT; 0.35 and 0.62, respectively, Fig. 1g). $\mu ^{\prime}_{{S,m}}$ explained 35 % of light-and temperature-dependent bleaching variance Δ*PE* for HL and HT (r^2^ = 0.35, *p* = 0.07, Additional file 3: Figure S3b). The ecological relevance of high- and low-$\mu ^{\prime}_{{S,m}}$ remains to be fully understood, but current evidence points to very distinct ecological strategies. Skeleton deposited by corals is made of calcium carbonate nanograins (about 50–200 nm diameter) (e.g., [35]) that govern the scattering properties of the skeleton and present a fractal micro-morphology (i.e., structures between 30 and 1000 nm that have a similar degree of compactness [18]) likely reflective of their growth strategy and skeletogenesis. Corals with higher rates of linear extension, rather than skeletal infilling (typical of branching species), often have the lowest $\mu ^{\prime}_{{S,m}}$ values and are typically thin branching, as opposed to corals with high-$\mu ^{\prime}_{{S,m}}$ which often have higher skeletal density and are massive or thick branching [18]. A prior study of light scattering and skeletal fractality in 150 coral skeletons representing 94 coral taxa demonstrated that high and low-$\mu ^{\prime}_{{S,m}}$ corals are important species in a variety of ecosystems. For example, *S. hystrix* and *S. pistillata*, two representatives of the Pocilloporidae family with low-$\mu ^{\prime}_{{S,m}}$, can be frequently found in Central, Eastern, and Western Indo-Pacific reefs, while *Porites lobata* and *Orbicella annularis* of the Poritidae and Merulinidae families with high-$\mu ^{\prime}_{{S,m}}$ are important species in Eastern Indo-Pacific and Caribbean reefs, respectively.

This study focused on the light scattering within skeleton and light absorbed by *Symbiodinium in hospite*, but did not evaluate light scattering within coral tissue which has been shown to significantly modulate light availability to symbionts. Light scattering causes lateral redistribution within tissue and increases light availability to symbionts [19, 28] while host fluorescent pigments [33, 51] or tissue contraction [19, 52] may reduce light stress by regulating light exposure and travel within tissue. Direct evidence for the optical feedback hypothesis would require in vivo measurements of *Symbiodinium* light-absorption rates as the coral undergoes bleaching and separation of skeleton-dependent effects, which has proven to be a technical challenge. However, combining the model of light absorbed by *Symbiodiniumin hospite* developed in this study and light available to *Symbiodinium* within the coral tissue measured with light microsensors [19, 28, 32] will improve models of the optics of intact corals. In fact, integrating within-tissue light scattering with skeletal scattering will allow for a comprehensive evaluation of the mechanisms of light scattering by skeleton and tissue in modulating light to symbionts and their role in bleaching response.

### Factors that did not influence bleaching response

Neither *R*_*S*_ (Fig. 3b, c, f; Additional file 4: Figure S4; LMM *p* > 0.15) nor $\mu^{\prime}_{S}$ (r^2^ = 0.02 for Δ*PE p* > 0.5) were significantly correlated with the severity of bleaching response. Light reflectance in coral skeletons is a complex process, and an important distinction must be made between $\mu ^{\prime}_{{S,m}}$, which governs short-path light transport in the superficial skeletal layer, and the reduced scattering coefficient of the entire skeletal material, $\mu^{\prime}_{S}$. Short-path transport is primarily driven by scattering of nanograins and fiber bundles of the top ~100 µm and is less influenced by larger structures such as overall morphology of corallites, optical properties of deeper skeletal material, or absorption. Although *R*_*S*_ includes the effect of short-path light-transport, it is primarily determined by $\mu^{\prime}_{S}$, absorption, and overall coral morphology (see Additional file 6: Text S1.1). In agreement with this, $\mu^{\prime}_{S}$ assessed for the ten coral species in the present study was not a good predictor of bleaching response. This difference between $\mu ^{\prime}_{{S,m}}$ and $\mu^{\prime}_{S}$ was also observed for 22 coral taxa [18]; modeling of the effect of $\mu ^{\prime}_{{S,m}}$ on bleaching showed that the rate of increase of light enhancement with decrease of absorbers (microspheres modeling symbiont pigments)is inversely dependent on $\mu ^{\prime}_{{S,m}}$. Although this model couldn’t be applied to test the effect of $\mu^{\prime}_{S}$ on bleaching in thin (1–2 mm) polished skeletal laminae, integrating sphere measurements of $\mu^{\prime}_{S}$ for 22 coral taxa showed no correlation with their bleaching susceptibility, further supporting observations of the current study [18].

Skeletal *R*_*S*_ and $\mu ^{\prime}_{{S,m}}$ affect coral physiology through two opposing light-modulation pathways: $\mu ^{\prime}_{{S,m}}$ is inversely related to the *rate* of light amplification increase [18], *R*_*S*_ is directly related to total light amplification [15, 17, 30]. Both $\mu ^{\prime}_{{S,m}}$ and *R*_*S*_ have the potential to increase light availability to symbionts [15–18, 29] and exacerbate the bleaching response [15, 18]. While our results identified a connection between $\mu ^{\prime}_{{S,m}}$ and bleaching response, no correlation between *R*_*S*_ or $\mu^{\prime}_{S}$ and *F*_*v*_*/F*_*m*_ was detected. Parallel to the hypothesis that the threshold for bleaching is determined by temperature increase rate [53], the threshold for light-enhanced bleaching appears to be determined by light-increase rate (associated with $\mu ^{\prime}_{{S,m}}$) rather than the total light (associated with *R*_*S*_).

Even though *Symbiodinium* thermotolerance (physiotype) has been shown to increase holobiont thermotolerance (1–2 °C [54]) in a pattern that dominates current theory explaining differential bleaching susceptibility [2, 11–14], it was not associated with bleaching response in these experiments. While three associations had similar tolerances and susceptibilities, the most thermotolerant symbionts (D1, D1a, and C8a) were hosted by the most bleaching susceptible corals (*P. damicornis, S. hystrix and S. pistillata* [5, 6, 18]), and the most thermosensitive symbionts (B1, C3v, and C3u) were hosted by the most bleaching resistant corals (*D. labyrinthiformis, Goniopora* sp., and *F. favus* [5, 6, 18]); providing an opportunity to detect effects of symbiont physiotypes. Similar to recent evidence that differential bleaching susceptibility cannot be explained by symbiont thermotolerance alone [10, 55, 56], no positive correlation between *Symbiodinium* thermotolerance and *F*_*v*_*/F*_*m*_ or *Q*_*m*_ was detected (LMM, p > 0.5 and 0.05, respectively, Fig. 3d, e, g; Additional file 5: Figure S5). While thermotolerance is demonstrable within a single life-stage of an individual species [11] or in isolation [43], it is generally context-dependent within the physiological and physical properties of the coral host [8, 12, 55, 57] and environment [12, 58].

We evaluated potential confounding factors of $\mu ^{\prime}_{{S,m}}$: in the absence of stress, low-$\mu ^{\prime}_{{S,m}}$ corals had lower *Symbiodinium* density, higher chlorophyll, and higher *F*_*v*_*/F*_*m*_ (t test, all *p* < 0.001), but these factors were not found to significantly associate with differential bleaching severity among the ten studied coral species. While this study cannot rule out the existence of other unknown potential confounders that may correlate with $\mu ^{\prime}_{{S,m}}$ and better explain the differential bleaching severity among these species, we have proposed a mechanism that explains the association of $\mu ^{\prime}_{{S,m}}$ with differential bleaching severity.

## Conclusions

Skeletal scattering was predictive of beaching susceptibility in these experiments and, if these results are representative of wider patterns, then they indicate that skeletal scattering is one of the key determinants of differential bleaching susceptibility. While symbionts associated with low-$\mu ^{\prime}_{{S,m}}$ corals may receive less total light from their skeletons, they are predicted to experience a higher rate of (skeletally-derived) light increase once bleaching is initiated and absorbing bodies are lost; further precipitating the bleaching response. While $\mu ^{\prime}_{{S,m}}$ explained 62 % of the light-dependent variance in bleaching response, it was a poor predictor of the temperature-dependent variance and it explained 35 % of the light- and temperature-dependent bleaching variance. Therefore, the remaining variance must be explained by other determinants of bleaching susceptibility. Symbiont phylotype can affect host physiology, holobiont fitness, and bleaching susceptibility [12, 54, 59]; higher symbiont densities per coral cell increase the risk of coral bleaching [55]; coral morphological and physiological properties modulate available light to the symbiont, determine early stress responses, and regulate symbiont photosynthetic demand for CO_2_ [8, 30, 57]; within-tissue light scattering increases light availability to symbionts [19, 28] and may reduce the threshold for bleaching. The challenge now is to discern the contribution of the key determinants of bleaching susceptibility in order to identify the most effective management and remediation strategies to protect the remaining diversity of coral-*Symbiodinium* associations in a changing climate.

## Methods

The predictions of the optical feedback hypothesis were experimentally assessed by monitoring the effects of differential $\mu ^{\prime}_{{S,m}}$ on the dynamics of bleaching response for a diverse set of 10 corals and modelling skeleton-dependent light absorption by *Symbiodinium* from experimentally measured values of coral reflectance (*R*_*H*_ during bleaching and *R*_*S*_ of bare skeletons). Low-$\mu ^{\prime}_{{S,m}}$ corals should experience increased rates and severities of bleaching-response as indicated by dynamically decreased density of *Symbiodinium* (*Δρ*) and/or photosynthetic pigments per *Symbiodinium* cell (*Δ*Chl *a*) and increased skeletal exposure (*ΔR*_*H*_), increased rates and severities of light stress on the *Symbiodinium* which remain *in hospite* as indicated by photosynthetic performance (*ΔF*_*v*_*/F*_*m*_ and *ΔQ*_*m*_) and increased light absorption (Δ*I*_*a*2_/*ρ*). Because of the diversity of corals employed in this study, we assessed alternative factors (known and hypothesized) for their contribution to experimental bleaching responses, including physical properties of the host (skeletal reflectance and coral tissue thickness), and differences in *Symbiodinium* phylotype thermotolerance known from the historical record.

### Coral host and *Symbiodinium* types

Colonies were prescreened for diversity of $\mu ^{\prime}_{{S,m}}$, *R*_*S*_, and *Symbiodinium* thermotolerance (Table 1). Coral were selected from live collections of Shedd Aquarium, Chicago, IL, USA (*P. damicornis*, *S. hystrix*, *S. pistillata*, *T. reniformis*, *M. foliosa*, and *M. digitata* originating from the Indo-pacific; and *D. labyrinthiformis* originating from Key West, Florida, USA) or obtained through A&M Aquatics, Lansing, MI, USA (*Goniopora* sp., *F. favus,* and *Merulina* sp. originating from Jakarta, Indonesia or Fiji). All corals were property of Shedd Aquarium, who granted research approval through their institutional review board; none of the coral species are listed as endangered or threatened by the US Endangered Species Act. All colonies were acclimated under control conditions (26 °C and 83.1 ± 1 μmol quanta m^−2^ s^−1^ on a 10/14 h light/dark cycle) 2–4 weeks prior to fragmentation and recovered 3–5 weeks under the same conditions. Ramets were created by cutting parent colonies into 32 ~ 1.5 cm^2^ explants with a wet tile-saw primed with artificial sea water (37 ^0^/_00_ salinity) and mounted to natural stone tiles using aquarium epoxy or ethyl 2-cyanoacrylate. Mounted corals where evenly distributed among four sectors in two aquaria. The tissue thickness of eight of the ten colonies were measured directly (reported as the mean of ten measurements) from size-standardized digital photos (using ImageJ version 1.47; NIH) of live colonies when cut in cross section, while the tissue thickness of *D. labyrinthiformis* and *Goniopora* sp. were estimated from published measurements (Table 1).

Holobiont tissue was scraped from skeletons and nucleic acids were extracted using standard protocols [60]. Identification markers [*Symbiodinium* nuclear internal transcribed spacer region 2 (ITS2) and chloroplast 23S ribosomal DNA (23S rDNA), and Scleractinia mitochondrial cytochrome oxidase I (COI), cytochrome b (CytB), and nuclear ITS] were selectively amplified by polymerase chain reaction (PCR) using standard reagents and the primers and annealing temperatures listed in Additional file 7: Table S1a and Additional file 6: Text S1.2. PCR products were separated by gel electrophoresis and directly sequenced using the amplification primers and identified by similarity (i.e., BLAST search) with GenBank accessions (Table 1). All DNA sequences created in this study are accessioned in GenBank as documentation of identity (Additional file 8: Table S1b). Morphological identification [61] was used for coral taxa novel to Genbank (Table 1). Thermotolerance of *Symbiodinium* phylotypes was designated following previous research (Table 1).

### Microscopic reduced light-scattering coefficient, $\mu ^{\prime}_{{S,m}}$

Microscopic-skeletal scattering ($\mu ^{\prime}_{{S,m}}$) was measured using low-coherence enhanced backscattering spectroscopy (LEBS) on corals cleaned with pressurized artificial seawater, soaked for <12 h in 3 % sodium hypochlorite, rinsed, and dried. We focused on short-propagating photons from the upper ~100–200 microns of skeletons to reduce the effects of ‘bulk-scattering’ properties [18]. The LEBS instrument has been previously described [62–64], and its application to coral ecology demonstrated [18]; but briefly, this method uses constructive interference of photons observed as an angular intensity cone centered in the backscattering direction to measure microscopic-scattering through broadband partial spatial coherence illumination. The LEBS instrument uses linearly polarized collimated broadband illumination directed at the surface of a coral skeleton at 15º angle of incidence, and light backscattered by the coral is collected using a lens, a polarizer, and an imaging spectrograph coupled with a CCD camera. The camera records a matrix of light-scattering intensities, *I*_*LEBS*_(*θ*, *λ*), as a function of wavelength *λ* (450–700 nm) and backscattering angle *θ* (−5 to 5 degrees). The spatial coherence length of illumination, *Lsc*, was fixed at ~57 microns at 600 nm illumination. The reduced scattering coefficient of $\mu^{\prime}_{S}$ was measured on cleaned coral skeletons using the enhanced backscattering spectroscopy (EBS) method as previously described [64–66].

### Skeletal and holobiont reflectance (R_s_ and R_H_)

Holobiont reflectance, *R*_*H*_, is used to quantify bleaching: as *Symbiodinium* cell and photopigment density decrease, the skeleton becomes increasingly visible through host tissues and *R*_*H*_ increases [15, 16, 27, 29]. To prepare corals for *R*_*S*_ measurements, tissue was removed from ramets with their skeletons remaining attached to their tiles so that they could be returned to the same location and orientation as they were during the collection of *R*_*H*_ measurements. Preservation of the experimental conditions during measurement of *R*_*S*_ insured that the intensity and direction of downwelling incident light was maintained and that *R*_*S*_ would be comparable to *R*_*H*_. Tissue was removed (by pressurized water), and preserved for *Symbiodinium* and pigment density analysis, and cleaned (as above) prior to measurement of *R*_*S*_.

Reflectance, *R*_*H*_ and *R*_*S*_, were measured as spectral reflectance using an optical fiber (Thorlabs SFS200/220Y) attached to a spectrometer (Ocean Optics USB4000). This method uses the Lambertian nature of the diffusely reflected light to enable hand-held measurement. Radiant flux is independent of angle and distance for a flat Lambertian scattering surface, however coral surfaces are irregular and small signal variations occur in different fiber positions. To account for this variation, ten measurements were collected randomly across the geometry of the ramet for each time point and specimen. The fiber was held at a distance of 1–2 cm from the upper surfaces of the ramet, near normal to the illumination source, while simultaneously avoiding shading the interrogation spot. The aperture of the fiber and refractive index of the water determine the acceptance angle of light, therefore this method interrogates a 3–6 mm diameter spot which will include signal from polyp and coenosarc. Measurements were normalized to a white reflectance standard (PTFE, Ocean Optics) adjacent to each ramet. The raw spectral reflectance for *R*_*S*_ and *R*_*H*_ was not further processed (e.g., by applying low-pass filters that smooth signal averages of high frequencies, making the spectra appear less variable), as the signal to noise ratio is sufficiently high to distinguish changes in *R*_*H*_ during bleaching (Additional file 6: Text S1.1).

### Experimental design

The two experimental aquaria are 420 L (~25 cm depth) recirculating unidirectional (2.5–4 cm/s) baffled flumes, with the corals at ~15 cm depth. The illuminating arrays (high color temperature that approximates sunlight) are divided by suspended shades to allow independent control of light conditions in each half of each aquarium. Explants were assigned to light sectors (8 ramets of each coral species) and randomly distributed within a sector to acclimate. See Additional file 9: Figure S6, and Additional file 6: Text S1.3 for details.

Stress was induced in three treatments (control remained static) by increasing the temperature to 32.3 ± 0.5 °C (over 2 days) in one aquarium and light levels to 328.1 ± 4.3 µmol photons m^2^/s in half of both aquaria (dynamic photoinhibition has been observed at 200–400 µmol quanta m^−2^ s^−1^ [67] and a trial experiment showed chronic photoinhibition of these corals at >400 umol quanta m^−2^ s^−1^ with no increase in temperature). This established four conditions: (1) control temperature and control light (CT-CL: 26.2 ± 1 °C at 83 ± 1 µmol photons m^2^/s), (2) control temperature and high light (CT-HL: 26.2 ± 1 °C at 328 ± 4.3 µmol photons m^2^/s), (3) high temperature and control light (HT-CL: 32.3 ± 0.5 °C at 83 ± 1 µmol photons m^2^/s), and (4) high temperature and high light (HT-HL: 32.3 ± 0.5 °C at 328 ± 4.3 µmol photons m^2^/s) (Additional file 9: Figure S6). Ramets were assessed every second day for 10 days prior to stress induction and 11 days thereafter (Additional file 6: Text S1.3). Any ramets with necrotic tissue (1.3 % of replicates) were removed from the experiment. Bleaching response was evaluated by the dynamics of *Symbiodinium* and photopigment density, holobiont reflectance, and *Symbiodinium* photosynthetic performance.

### *Symbiodinium* photophysiology

*Symbiodinium* photosynthetic performance was assessed through pulse-amplitude modulation (PAM) chlorophyll fluorometry with a 1.5 mm diameter optical fiber and the following instrument settings: measuring intensity 6, saturation intensity 12, saturation width 0.6, and actinic light intensity 9. Induction curves were collected with the *F*_*0*_*ʹ*-mode (far-red light) activated and a delay of 40 s, a width of 20 s, and a length of 13 cycles (Additional file 6: Text S1.4). Dark-adapted yield of photosystem II (PSII) was measured (where *F*_*v*_/*F*_*m*_ = *F*_*m*_ − *F*_0_/*F*_*m*_) at 07:20–08:00 h (prior to sunrise) and induction curve analyses were performed at 09:00–13:00 h (at peak irradiance). Data for induction curves were collected through the steady state of *Fʹ* and *F*_*m*_*ʹ* and effective quantum yield (*Φ*_*PSII*_ = *F*_*m*_*ʹ* − *Fʹ*/*F*_*m*_*ʹ*), non-photochemical quenching (*Φ*_*NPQ*_ = *Fʹ*/*F*_*m*_*ʹ* − *Fʹ*/*F*_*m*_) and non-regulated heat dissipation (*Φ*_*NO*_ = *Fʹ*/*F*_*m*_) were calculated from steady state measurements where *Φ*_*PSII*_ + *Φ*_*NPQ*_ + *Φ*_*NO*_ = 1 [42]. Photochemical efficiency, *F*_*v*_*/F*_*m*_, was used as a metric of bleaching response and has repeatedly been shown to decrease during bleaching [25, 43]. *Symbiodinium* exhibit *Φ*_*PSII*_ oscillations when light absorption exceeds photochemistry [40], which is measured here as maximum excitation pressure over photosystem II, or *Q*_*m*_ = 1 − [(*Φ*_*PSII at peak light*_)/(*F*_*v*_*/F*_*m at dawn*_)] [40, 41] (Additional file 6: Text S1.5).

### *Symbiodinium* and photosynthetic pigment density

*Symbiodinium* cells were collected using pressurized seawater and the resulting slurry was concentrated by centrifugation before being divided into aliquots for hemocytometer cell counts (Additional file 6: Text S1.6) and high-performance liquid chromatography (HPLC) analysis of photosynthetic pigment identities and concentrations (Additional file 6: Text S1.7) using established procedures and gradients [68]. Surface area estimation of skeletons (for normalizing cell counts) were estimation using the single-dip wax method [69].

### Statistical analysis

General linear model ANOVAs were performed in Minitab to test the effect of $\mu^{\prime}_{S}$_*m*_ on change in *Symbiodinium* cell and photosynthetic pigments density, *ΔR*_*H*_, *ΔF*_*v*_*/F*_*m*_, or *ΔQ*_*m*_. Hierarchical linear mixed models (LMM) were applied in Stata 11.2 to account for the repeated-measures design [70] to assess the overall effect of treatment (time, light, and temperature) on bleaching response in the 11-day experiment (Additional file 6: Text S1.8). These analyses focused on the effect of potential explanatory variables ($\mu^{\prime}_{S}$_*m*_, *R*_*S*_, and *Symbiodinum* thermotolerance) on photophysiological response (*F*_*v*_*/F*_*m*_ and *Q*_*m*_).

### Determining light-dependent and temperature-dependent bleaching effects

To determine the effect of light and temperature on bleaching separately, we used Taylor Series Expansion to mathematically isolate factors of interest (effect of light or temperature on temporal rates of *F*_*v*_*/F*_*m*_ decrease) and cancel out known and unknown confounders across conditions because the physical conditions of the live animal experiment cannot be made precisely identical across all ramets. For example, potential confounders such as differential tissue thickness and localized morphology-induced flow diversity among explants of the same colony could alter mass transfer across the diffuse boundary layer and affect bleaching response [16, 71–73]; these factors cannot be fully controlled among such a large number of ramets. However, they can be mathematically cancelled out from all conditions by subtracting the difference between temporal rates of *F*_*v*_*/F*_*m*_ decrease (*PE*) under control and stress conditions for each environmental factor (light or temperature).

To determine the light-dependent bleaching effect, we examined the difference between *PE* under control and high light conditions. For a given ramet *i*, the temporal rate of *F*_*v*_*/F*_*m*_ decrease, $PE_{i} = \frac{{\varDelta \left( {{{F_{V} } \mathord{\left/ {\vphantom {{F_{V} } {F_{m} }}} \right. \kern-0pt} {F_{m} }}} \right)_{i} }}{\varDelta t}$, where *t* is time after the initiation of bleaching, was expressed as the first order Taylor expansion over temperature, light intensity, and potential confounding (including unknown) factors:

1 $$ PE_{i} \left( {T,I} \right) = X_{i} + \Updelta T\left. {\frac{{\partial PE_{i} }}{\partial T}} \right|_{{T_{1} ,I_{1} }} + \Updelta I\left. {\frac{{\partial PE_{i} }}{\partial I}} \right|_{{T_{1} ,I_{1} }} , $$

where Δ*T* is the difference between experimental temperature *T* and control *T*_1_, Δ*I* is the difference between experimental light intensity *I* and control *I*_1_, and *X*_*i*_ accounts for all other conditions (e.g., localized flow rates, without assuming that they are identical across ramets). To mathematically isolate the effect of light from temperature and confounding factors, *PE* values for corals exposed to CL (*I* = *I*_1_ ≡ *I*_*CL*_) were subtracted from corals exposed to HL (*I* = *I*_2_ ≡ *I*_*HL*_) for either control (*T* = *T*_1_) or high (*T* = *T*_2_) temperature:

2 $$ \varDelta PE_{i} \left( {T_{j} } \right) = PE_{i} \left( {T_{j} ,I_{HL} } \right) - PE_{i} \left( {T_{j} ,I_{CL} } \right) = \left( {I_{HL} - I_{CL} } \right)\left. {\frac{{\partial PE_{i} }}{\partial I}} \right|_{{T_{j} ,I_{CL} }} \propto \left. {\frac{{\partial^{2} \left( {{{F_{V} } \mathord{\left/ {\vphantom {{F_{V} } {F_{m} }}} \right. \kern-0pt} {F_{m} }}} \right)_{i} }}{\partial t\partial I}} \right|_{{T_{j} }} , $$

where index *j* indicates either high (*j* = 2) or control (*j* = 1) temperature environment. In the first order approximation, this differential quantity Δ*PE*_*i*_ is independent of factors not directly related to illumination.

Similarly, to mathematically isolate the effect of temperature from light and confounding factors (temperature-dependent bleaching effect), *PE* values for corals exposed to CT were subtracted from corals exposed to HT:

3 $$ \varDelta PE_{i} \left( {I_{j} } \right) = PE_{i} \left( {T_{HT} ,I_{j} } \right) - PE_{i} \left( {T_{CT} ,I_{j} } \right) = \left( {T_{HT} - T_{CT} } \right)\left. {\frac{{\partial PE_{i} }}{\partial T}} \right|_{{T_{CT} ,I_{j} }} \propto \left. {\frac{{\partial^{2} \left( {{{F_{V} } \mathord{\left/ {\vphantom {{F_{V} } {F_{m} }}} \right. \kern-0pt} {F_{m} }}} \right)_{i} }}{\partial t\partial T}} \right|_{{I_{j} }} , $$

where index *j* indicates either HL (*j* = 2) or CL (*j* = 1) environment.

*ΔPE* was analyzed as a function of potential explanatory variables (potential determinants of bleaching response; $\mu^{\prime}_{S}$_*m*_, *R*_*S*_, tissue thickness, and *Symbiodinium* thermotolerance) and confounders of $\mu^{\prime}_{S}$_*m*_ (initial *F*_*v*_*/F*_*m*_, initial *Symbiodinium* and chl *a* density), thereby removing differences in bleaching response that are not explicitly related to light.

### Skeleton-dependent light absorption model

We developed a novel model of *Symbiodinium* light absorption, which, in comparison to existing models, accounts for skeleton-driven absorption and multiple reentry effects. Incident light absorption by *Symbiodinium* (fraction *I*_*a*_) can be viewed as the result of absorption of downwelling light (fraction *I*_*a*1_ of the incident light) and skeleton-dependent absorption (fraction *I*_*a*2_ = *I*_*a*_ − *I*_*a*1_) of light reflected by the skeleton [15–17]. Light that is not absorbed in the first pass (1 − *I*_*a*1_) can be reflected by the skeleton back into the tissue, lost to skeletal absorption, or diffusely scattered out of the colony [17, 19, 27, 28]. This process may involve multiple passes of light through tissue due to multiple reentries of unabsorbed light back into the skeleton and subsequent reflections by the skeleton [15, 17]. Because direct quantification of light absorption by pigments in live corals is not currently possible, we developed an empirical model relating *I*_*a*1_ and *I*_*a*2_ with experimentally measurable parameters *R*_*S*_ and *R*_*H*_.

Starting with balance equations for *R*_*H*_ and *I*_*a*_, we solve for *I*_*a*1_ and *I*_*a*2_ [see Additional file 6: Text S1.9 for detailed derivation using equations (4) through (7)]:

4 $$R_H=R_1(1-I_{a1})(1-a_{2}),$$

5 $$ I_{a1} = \frac{1}{2\alpha }\left( {1 + \alpha - \sqrt {\left( {1 + \alpha } \right)^{2} - 4\alpha \left( {1 - \beta R^{\prime}} \right)} } \right) , $$

6 $$I_a=I_{a1}+(1-I_{a1}){R_1}{a_2}+(1-I_{a1})\gamma (R_s-R_1),$$

7 $$ I_{a2} = I_{a} - I_{a1} = \left( {1 - I_{a1} } \right)\left( {\frac{\alpha }{\beta }I_{a1} + \gamma \frac{\beta - 1}{\beta }} \right)R_{S} , $$

where *R*′ = *R*_*H*_/*R*_*S*_, *β* = *R*_*S*_/*R*_1_, *α* = *a*_2_/*I*_*a*1_ with *R*_*1*_ the fraction of unabsorbed light that is leaving the holobiont after being reflected by the skeleton back into tissue including all reentries and *a*_2_ the fraction of this reflected light that is absorbed by the pigments in the tissue, and *γ* is the fraction of light that is absorbed by tissue through processes other than *I*_*a*1_ or *a*_2_ divided by (*R*_*S*_ − *R*_1_).

Coefficients *α*, *β*, and *γ* depend on coral morphology, its optical properties, and the concentration of absorbing pigments in tissue (see Additional file 6: Text S1.9 for detailed explanation). Coefficient *α*(>1) describes the amplification of light absorption due to elongation of light paths through the tissue caused by diffuse skeletal reflection of unabsorbed downwelling light, which is why α increases as the concentration of absorbing pigments decreases. Coefficients *β* and *γ* are related to the non-flatness of the skeleton and account for the reentry effect. In the special case of no reentry (flat coral model), $\beta = \gamma = 1$ and $1 < \alpha \le 2$. Non-flat skeletons can create *α* > 2 due to multiple reentry [15] and $\beta > 1$ and *γ* < 1 for non-flat geometries. If reentry is neglected, Eq. 7 for *I*_*a*_ converges to the solution that has been conventionally used to estimate the light absorption based on holobiont and skeletal reflectances [15, 29, 49], $I_{a} \approx 1 - R^{\prime}$, if one of the following two conditions is satisfied: *I*_*a*2_ can be neglected (most of the absorption is due to the downwelling light) or *R*_*S*_ = 1. Even though *α*, *β*, and *γ* depend on concentration and the optical properties of the skeletons, the model can still be used to estimate the range of *I*_*a*1_ and *I*_*a*2_. Indeed, *I*_*a*2_ increases with α (e.g., as symbionts leave). Thus, we can obtain the lower bound on *I*_*a*2_ by using Eqs. (5) and (7) with *α* = *β* = *γ* = 1.

## Consent to publish

Express written informed consent has been granted for publication of Figure S6.

## Availability of data and materials

All supporting data are submitted to GenBank (accession numbers KF492657–KF492693) or are included as additional files.

## Additional files

10.1186/s12898-016-0061-4 Dynamics of holobiont reflectance (*R*_*H*_). Panels a–f are aligned into columns defined by light (broken line in a) and temperature (dotted line in a) conditions (described in Figure S1). Response of an exemplar low-$\mu ^{\prime}_{{S,m}}$ coral (*S. pistillata*) through (b) time series photos of explants, (c) spectral R_H_, and (f) means (black line) and standard errors of the 10 random measurements collected to estimate *R*_*H*_ normalized to its skeleton reflectance at 675 nm. Response of an exemplar high-$\mu ^{\prime}_{{S,m}}$ coral (*M. digitata*) through (d) time series photos of coral explants, (e) spectral R_H_ and (f) means (gray line) and standard errors of the 10 random measurements collected to estimate *R*_*H*_ normalized to its skeleton reflectance at 675 nm. Spectral skeletal reflectance (*R*_*S*_) in panels c and e shown to contextualize *R*_*H*_ with the limit of *R*_*S*_ values in the visible spectrum where photopigments have substantial absorption (e.g., 675 nm, chlorophyll *a* absorption peak); for wavelengths > 700 nm, the limit of *R*_*H*_ may be greater than *R*_*S*_. As corals bleached and less than 10 % of symbionts remained associated with the host, *R*_*H*_ approached the values of *R*_*S*_.
10.1186/s12898-016-0061-4 Dynamics of bleaching response variables for corals grouped by $\mu ^{\prime}_{{S,m}}$. Panels (a–h) aligned into columns defined by experimental conditions (CT-CL: control temperature [26 °C] and light [83 μmol quanta m^−2^ s^−1^], CT-HL: control temperature and high light [328 μmol quanta m^−2^ s^−1^], HT-CL: high temperature [32 °C] and control light, and HT-HL: high temperature and high light; shaded areas are control). Responses of high- (gray line) and low-$\mu ^{\prime}_{{S,m}}$ (black line) corals for (a) holobiont reflectance (dashed lines are the corresponding post-experiment skeletal reflectance), (b) *Symbiodinium* cell density, (c) chlorophyll *a* density per *Symbiodinium* cell, (d) maximal photosynthetic efficiency, (e) effective quantum yield of photosystem II, (f) excitation pressure over photosystem II, (g) non-photochemical quenching, and (h) non-regulated heat dissipation. All error bars are standard error.
10.1186/s12898-016-0061-4 $\mu ^{\prime}_{{S,m}}$ and temperature- and light-induced bleaching response. $\mu ^{\prime}_{{S,m}}$-specific temporal rate of *F*_*v*_ */F*_*m*_ change (Δ*PE* ∼ Δ^2^(*F*_*V*_/*F*_*M*_)/(Δ*t*Δ*I*)) after stress-initiation is expressed as (a) the difference between CT and HT conditions (Eq. 3) for corals exposed to HL (filled circles; *p* = 0.22) and CL (open circles; *p* = 0.44), isolating the effect of temperature on bleaching response, and (b) *ΔPE* for HL and HT conditions (*p* = 0.07), where both temperature- and light-dependent bleaching response is evaluated. Although $\mu ^{\prime}_{{S,m}}$ predicts light-dependent bleaching (r^2^ = 62.3 and *p* = 0.007, Fig. 1g), it is a weak predictor of temperature-dependent bleaching and light- and temperature-dependent bleaching.
10.1186/s12898-016-0061-4 Dynamics of bleaching response variables for corals grouped by skeletal reflectance (*R*_*S*_). Panels (a–h) aligned into columns defined by experimental conditions (described in Figure S3). Responses of high- (gray line) and low-*R*_*S*_ (black line) corals for (a) holobiont reflectance (dashed lines are the corresponding post-experiment skeletal reflectance), (b) *Symbiodinium* cell density, (c) chlorophyll *a* density per *Symbiodinium* cell, (d) maximal photosynthetic efficiency, (e) effective quantum yield of photosystem II, (f) excitation pressure over photosystem II, (g) non-photochemical quenching, and (h) non-regulated heat dissipation. All error bars are standard error.
10.1186/s12898-016-0061-4 Dynamics of bleaching response variables for corals grouped by *Symbiodinium* thermotolerance (*Symb*_thermo_). Panels (a–h) aligned into columns defined by experimental conditions (described in Figure S3). Responses of high- (gray line) and low-*Symb*_thermo_ (black line) corals for (a) holobiont reflectance (dashed lines are the corresponding post-experiment skeletal reflectance), (b) *Symbiodinium* cell density, (c) chlorophyll *a* density per *Symbiodinium* cell, (d) maximal photosynthetic efficiency, (e) effective quantum yield of photosystem II, (f) excitation pressure over photosystem II, (g) non-photochemical quenching, and (h) non-regulated heat dissipation. All error bars are standard error.
10.1186/s12898-016-0061-4 Supporting Text. Supporting methods (1) and supporting references (2).
10.1186/s12898-016-0061-4 Nucleotide sequencing. Primers [79–82] and annealing temperatures used for polymerase chain reaction amplification and nucleotide sequencing.
10.1186/s12898-016-0061-4 Nucleotide sequencing. GenBank accession numbers of genes sequenced in this study.
10.1186/s12898-016-0061-4 Experimental setup. High temperature aquarium encompassing the HT-CL and HT-HL conditions on day 11 of the bleaching experiment (a); black divider separating light arrays, flow baffles, and mounted corals can be seen. Collecting PAM measurements in the control temperature aquarium within the CT-CL condition (CT-HL on the opposite side of black divider) on day 11 of the bleaching experiment (b); positioning of the PAM instrument probes above the coral explants mounted on stone tiles can be seen. Close up of probe holder (custom-machined acrylic block that ensures probes are returned to each explant in the same three-dimensional geometry as previous measurements) supported at a 23° angle by square PVC post (gray) attached to the coral-mounting tile (c); probes are (left to right) temperature, O_2_ (data not reported), and PAM fiber optic immobilized in a black PVC tube. Control temperature aquarium encompassing the CT-CL and CT-HL conditions (HT aquarium in background) during acclimation period prior to prescreening and fragmentation (d); photograph taken before installation of the flow baffles and black divider separating light arrays. Hand-held optical fiber attached to a spectrometer to measure *R*_*H*_ (and *R*_*S*_ of cleaned skeleton) with white reflectance standard visible in the background (e). Consent to publish these images has been documented.

## Abbreviations

Chl *a*: chlorophyll *a* density
COV: coefficient of variation
CT-CL: control temperature-control light
CT-HL: control temperature-high light
EBS: enhanced backscattering spectroscopy
*Fʹ*: fluorescence yield in actinic light
*F*_*m*_: maximum fluorescence yield
*F*_*m*_*ʹ*: maximum fluorescence yield in actinic light
*F*_*v*_: maximum variable fluorescence yield
*F*_*v*_*/F*_*m*_: photochemical efficiency
*F*_*0*_: minimum fluorescence yield
*F*_*0*_*ʹ*: minimum fluorescence yield in light-acclimated state
HT-CL: high temperature-control light
HT-HL: high temperature-high light
*I*: light intensity
*I*_*a*_: *Symbiodinium* light-absorption
*I*_*a*1_: skeleton-independent light-absorption
*I*_*a*2_: skeleton-dependent light-absorption
LEBS: low-coherence enhanced backscattering spectroscopy
LMM: linear mixed models
PAM: pulse-amplitude modulation chlorophyll fluorometry
PE: temporal rate of *F*_*v*_*/F*_*m*_ (photochemical efficiency) reduction
PSII: photosystem II
*Q*_*m*_: maximum-excitation pressure over photosystem II
*R*_*H*_: holobiont reflectance
*R*_*S*_: diffuse reflectance of light from coral skeletons
*T*: temperature
α: amplification of light absorption due to elongation of light paths through the tissue caused by diffuse skeletal reflection of unabsorbed downwelling light
β: accounts for the reentry effect
γ: is the fraction of light that is absorbed by tissue through processes other than *I*_*a*1_ or *a*_2_ divided by (*R*_*S*_ − *R*_1_)
$\mu ^{\prime}_{{S,m}}$: microscopic reduced-scattering coefficient or the inverse of the distance a short-path length photon travels before randomization
$\mu^{\prime}_{S}$ or bulk-$\mu^{\prime}_{S}$: reduced-scattering coefficient or the inverse of the distance a photon travels before randomization
*ρ*: *Symbiodinium* cell density
*Φ*_*PSII*_: effective quantum yield of photosystem II
*Φ*_*NO*_: non-regulated heat dissipation
*Φ*_*NPQ*_: non-photochemical quenching
