# Supplementary material for: Skeletal light-scattering accelerates bleaching response in reef-building corals
Source: BMC Ecol. 2016 Mar 21;16:10. doi: 10.1186/s12898-016-0061-4 (PMC4800776; doi:10.1186/s12898-016-0061-4)
Supplement: Supplementary file 1 — 10.1186/s12898-016-0061-4 Dynamics of holobiont reflectance (R H). Panels a–f are aligned into columns defined by light (broken line in a) and temperature (dotted line in a) conditions (described in Figure S1). Response of an exemplar low-$\mu ^{\prime}_{{S,m}}$μS,m′ coral (S. pistillata) through (b) time series photos of explants, (c) spectral RH, and (f) means (black line) and standard errors of the 10 random measurements collected to estimate R H normalized to its skeleton reflectance at 675 nm. Response of an exemplar high-$\mu ^{\prime}_{{S,m}}$μS,m′ coral (M. digitata) through (d) time series photos of coral explants, (e) spectral RH and (f) means (gray line) and standard errors of the 10 random measurements collected to estimate R H normalized to its skeleton reflectance at 675 nm. Spectral skeletal reflectance (R S) in panels c and e shown to contextualize R H with the limit of R S values in the visible spectrum where photopigments have substantial absorption (e.g., 675 nm, chlorophyll a absorption peak); for wavelengths > 700 nm, the limit of R H may be greater than R S. As corals bleached and less than 10 % of symbionts remained associated with the host, R H approached the values of R S. [file 12898_2016_61_MOESM1_ESM.pptx]

## Slide 1
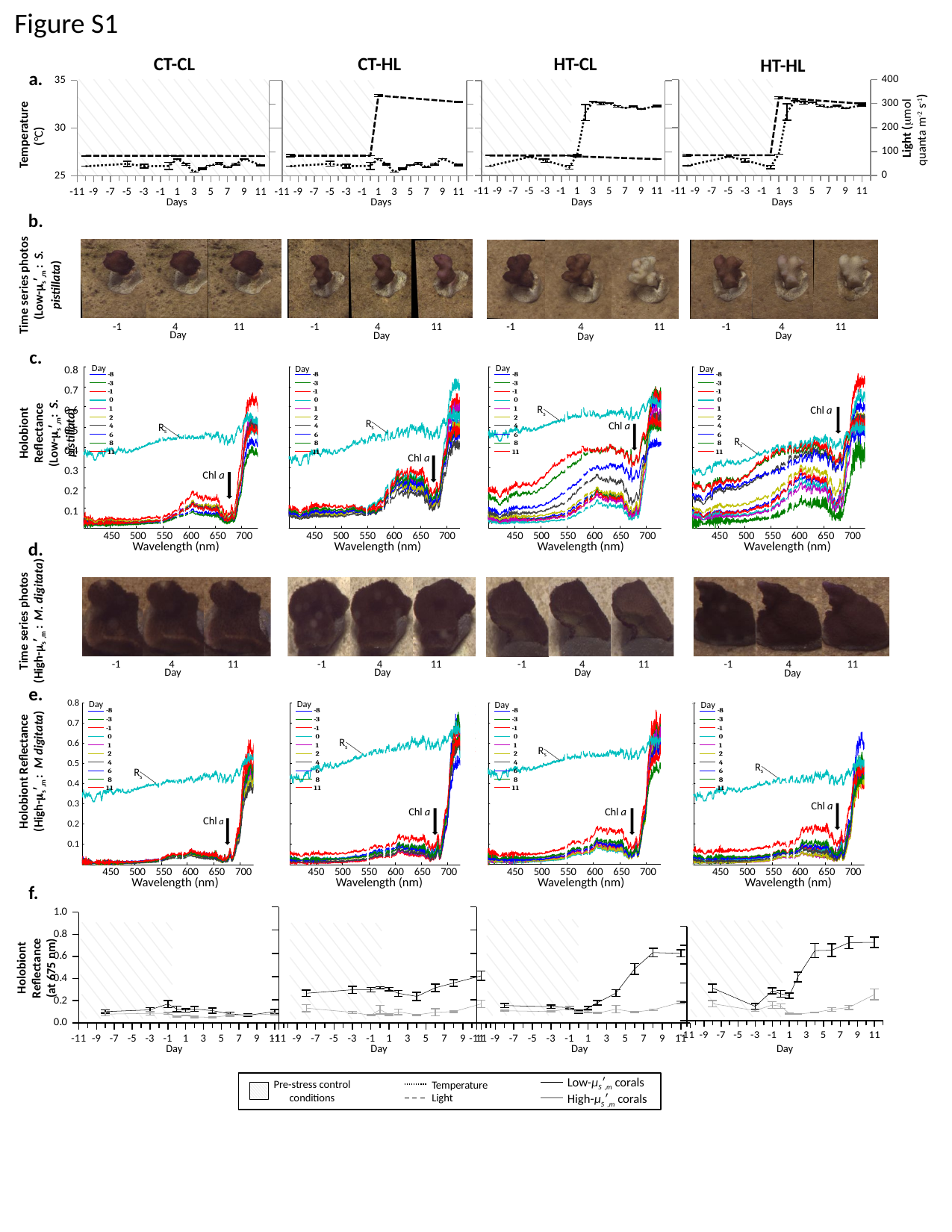

Figure S1
CT-CL
CT-HL
HT-CL
HT-HL
a.
### Chart
| Category | | |
|---|---|---|
### Chart
| Category | | |
|---|---|---|
### Chart
| Category | | |
|---|---|---|
### Chart
| Category | | |
|---|---|---|Light (μmol quanta m-2 s-1)
Temperature
(°C)
Days
Days
Days
Days
b.
Time series photos
(Low-μsʹ,m : S. pistillata)
-1 4 11
Day
-1 4 11
Day
-1 4 11
Day
-1 4 11
Day
c.
Day
Day
0.8
0.7
0.6
0.5
0.4
0.3
0.2
0.1
Day
Day
Holobiont Reflectance
(Low-μsʹ,m: S. pistillata)
Rs
Chl a
Rs
Chl a
Rs
Rs
Chl a
Chl a
450
500
550
600
650
700
450
500
550
600
650
700
450
500
550
600
650
700
450
500
550
600
650
700
d.
Wavelength (nm)
Wavelength (nm)
Wavelength (nm)
Wavelength (nm)
Time series photos
(High-μsʹ,m : M. digitata)
-1 4 11
Day
-1 4 11
Day
-1 4 11
Day
-1 4 11
Day
Holobiont Reflectance
(High-μsʹ,m : M digitata)
e.
0.8
0.7
0.6
0.5
0.4
0.3
0.2
0.1
Day
Day
Day
Day
Rs
Rs
Rs
Rs
Chl a
Chl a
Chl a
Chl a
450
500
550
600
650
700
450
500
550
600
650
700
450
500
550
600
650
700
450
500
550
600
650
700
Wavelength (nm)
Wavelength (nm)
Wavelength (nm)
Wavelength (nm)
f.
Holobiont Reflectance
(at 675 nm)
### Chart
| Category | | |
|---|---|---|
### Chart
| Category | | |
|---|---|---|
### Chart
| Category | | |
|---|---|---|
### Chart
| Category | | |
|---|---|---|Day
Day
Day
Day
Low-µSʹ,m corals
High-µSʹ,m corals
Pre-stress control conditions
Temperature
Light
