# Supplementary material for: Skeletal light-scattering accelerates bleaching response in reef-building corals
Source: BMC Ecol. 2016 Mar 21;16:10. doi: 10.1186/s12898-016-0061-4 (PMC4800776; doi:10.1186/s12898-016-0061-4)
Supplement: Supplementary file 2 — 10.1186/s12898-016-0061-4 Dynamics of bleaching response variables for corals grouped by $\mu ^{\prime}_{{S,m}}$μS,m′. Panels (a–h) aligned into columns defined by experimental conditions (CT-CL: control temperature [26 °C] and light [83 μmol quanta m−2 s−1], CT-HL: control temperature and high light [328 μmol quanta m−2 s−1], HT-CL: high temperature [32 °C] and control light, and HT-HL: high temperature and high light; shaded areas are control). Responses of high- (gray line) and low-$\mu ^{\prime}_{{S,m}}$μS,m′ (black line) corals for (a) holobiont reflectance (dashed lines are the corresponding post-experiment skeletal reflectance), (b) Symbiodinium cell density, (c) chlorophyll a density per Symbiodinium cell, (d) maximal photosynthetic efficiency, (e) effective quantum yield of photosystem II, (f) excitation pressure over photosystem II, (g) non-photochemical quenching, and (h) non-regulated heat dissipation. All error bars are standard error. [file 12898_2016_61_MOESM2_ESM.pptx]

## Slide 1
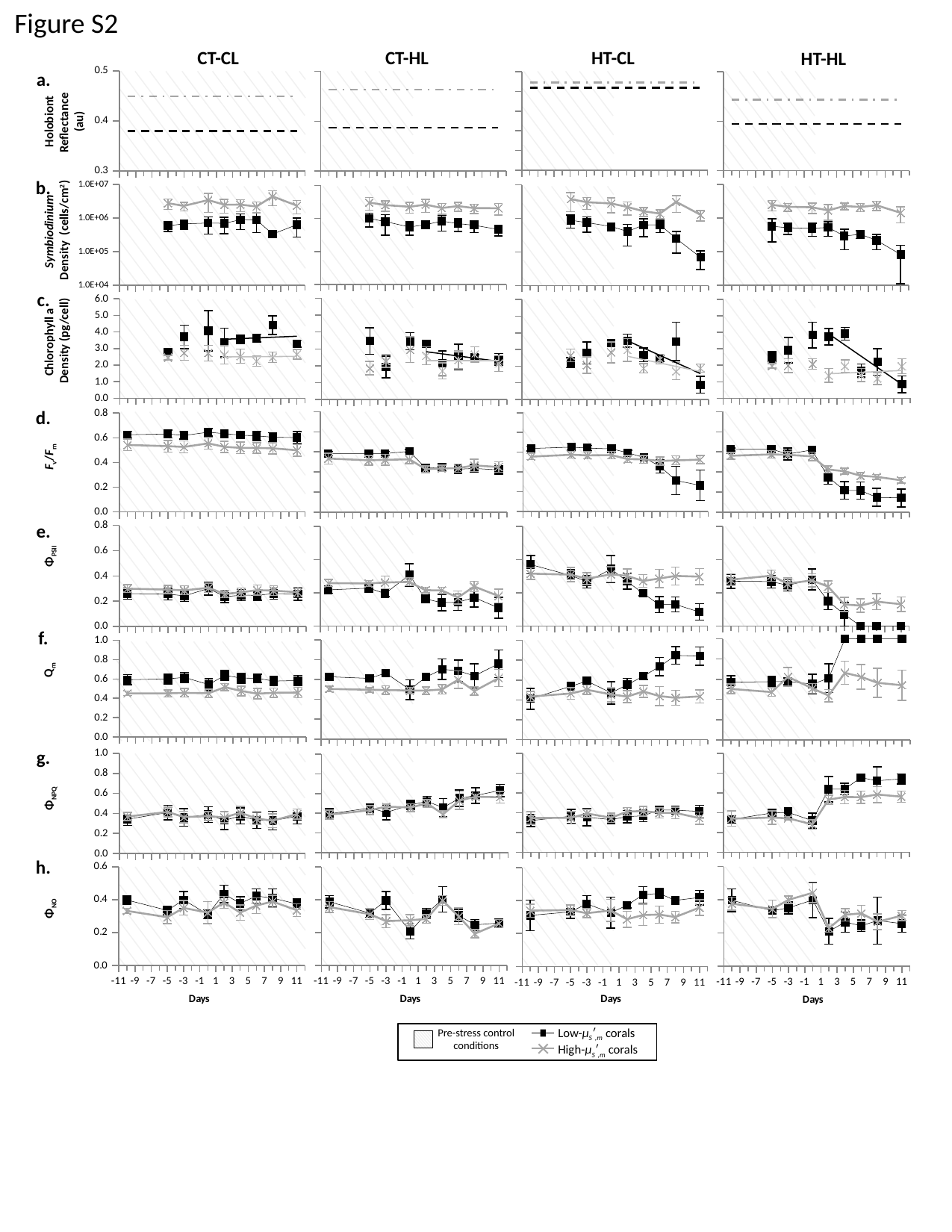

Figure S2
CT-CL
CT-HL
HT-CL
HT-HL
Holobiont Reflectance
(au)
a.
### Chart
| Category | low | high | | |
|---|---|---|---|---|
### Chart
| Category | low | high | | |
|---|---|---|---|---|
### Chart
| Category | low | high | | |
|---|---|---|---|---|
### Chart
| Category | low | high | | |
|---|---|---|---|---|Symbiodinium
Density (cells/cm2)
b.
### Chart
| Category | high mus' | low mus' |
|---|---|---|
### Chart
| Category | high mus' | low mus' |
|---|---|---|
### Chart
| Category | high mus' | low mus' |
|---|---|---|
### Chart
| Category | high mus' | low mus' |
|---|---|---|Chlorophyll a
 Density (pg/cell)
c.
### Chart
| Category | high mus' | low mus' | high mus' before | low mus' before |
|---|---|---|---|---|
### Chart
| Category | high mus' | low mus' | high mus' before | low mus' before |
|---|---|---|---|---|
### Chart
| Category | high mus' | low mus' | high mus' before | low mus' before |
|---|---|---|---|---|
### Chart
| Category | high mus' | low mus' | high mus' before | low mus' before |
|---|---|---|---|---|
### Chart
| Category | high mus' | low mus' |
|---|---|---|
### Chart
| Category | high mus' | low mus' |
|---|---|---|
### Chart
| Category | high mus' | low mus' |
|---|---|---|d.
### Chart
| Category | high mus' | low mus' |
|---|---|---|Fv/Fm
ΦPSII
e.
### Chart
| Category | high mus' | low mus' |
|---|---|---|
### Chart
| Category | high mus' | low mus' |
|---|---|---|
### Chart
| Category | high mus' | low mus' |
|---|---|---|
### Chart
| Category | high mus' | low mus' |
|---|---|---|f.
Qm
### Chart
| Category | high mus' | low mus' |
|---|---|---|
### Chart
| Category | high mus' | low mus' |
|---|---|---|
### Chart
| Category | high mus' | low mus' |
|---|---|---|
### Chart
| Category | high mus' | low mus' |
|---|---|---|g.
### Chart
| Category | high mus' | low mus' |
|---|---|---|
### Chart
| Category | high mus' | low mus' |
|---|---|---|ΦNPQ
### Chart
| Category | high mus' | low mus' |
|---|---|---|
### Chart
| Category | high mus' | low mus' |
|---|---|---|
### Chart
| Category | high mus' | low mus' |
|---|---|---|h.
### Chart
| Category | high mus' | low mus' |
|---|---|---|
### Chart
| Category | high mus' | low mus' |
|---|---|---|
### Chart
| Category | high mus' | low mus' |
|---|---|---|ΦNO
Low-µSʹ,m corals
High-µSʹ,m corals
Pre-stress control conditions
×
