# Supplementary material for: Skeletal light-scattering accelerates bleaching response in reef-building corals
Source: BMC Ecol. 2016 Mar 21;16:10. doi: 10.1186/s12898-016-0061-4 (PMC4800776; doi:10.1186/s12898-016-0061-4)
Supplement: Supplementary file 3 — 10.1186/s12898-016-0061-4 $\mu ^{\prime}_{{S,m}}$μS,m′ and temperature- and light-induced bleaching response. $\mu ^{\prime}_{{S,m}}$μS,m′-specific temporal rate of F v /F m change (ΔPE ∼ Δ2(F V/F M)/(ΔtΔI)) after stress-initiation is expressed as (a) the difference between CT and HT conditions (Eq. 3) for corals exposed to HL (filled circles; p = 0.22) and CL (open circles; p = 0.44), isolating the effect of temperature on bleaching response, and (b) ΔPE for HL and HT conditions (p = 0.07), where both temperature- and light-dependent bleaching response is evaluated. Although $\mu ^{\prime}_{{S,m}}$μS,m′ predicts light-dependent bleaching (r2 = 62.3 and p = 0.007, Fig. 1g), it is a weak predictor of temperature-dependent bleaching and light- and temperature-dependent bleaching. [file 12898_2016_61_MOESM3_ESM.pptx]

## Slide 1
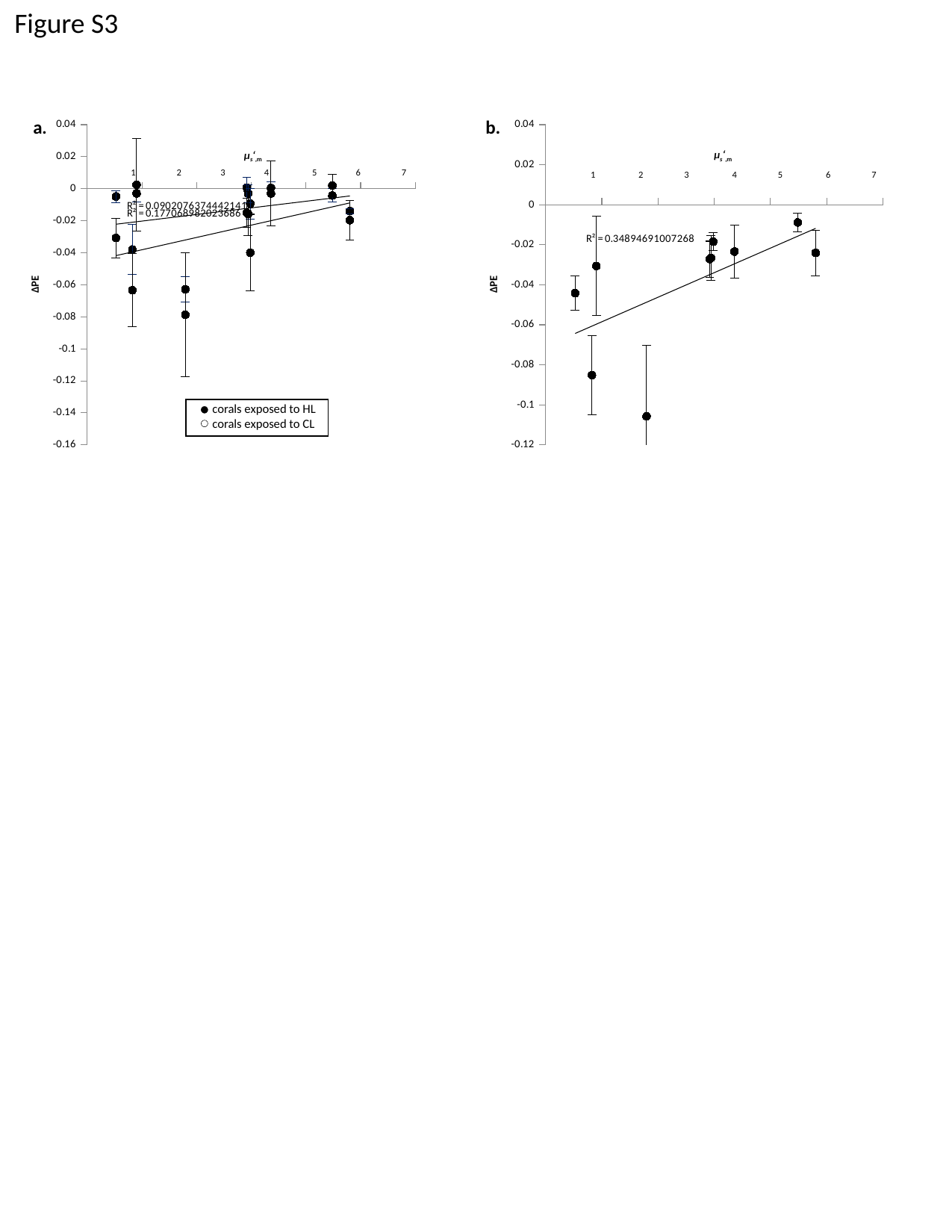

Figure S3
### Chart
| Category | | |
|---|---|---|
### Chart
| Category | |
|---|---|a.
b.
μs‘,m
μs‘,m
 1 2 3 4 5 6 7
 1 2 3 4 5 6 7
corals exposed to HL
corals exposed to CL
