# Supplementary material for: Skeletal light-scattering accelerates bleaching response in reef-building corals
Source: BMC Ecol. 2016 Mar 21;16:10. doi: 10.1186/s12898-016-0061-4 (PMC4800776; doi:10.1186/s12898-016-0061-4)
Supplement: Supplementary file 4 — 10.1186/s12898-016-0061-4 Dynamics of bleaching response variables for corals grouped by skeletal reflectance (R S). Panels (a–h) aligned into columns defined by experimental conditions (described in Figure S3). Responses of high- (gray line) and low-R S (black line) corals for (a) holobiont reflectance (dashed lines are the corresponding post-experiment skeletal reflectance), (b) Symbiodinium cell density, (c) chlorophyll a density per Symbiodinium cell, (d) maximal photosynthetic efficiency, (e) effective quantum yield of photosystem II, (f) excitation pressure over photosystem II, (g) non-photochemical quenching, and (h) non-regulated heat dissipation. All error bars are standard error. [file 12898_2016_61_MOESM4_ESM.pptx]

## Slide 1
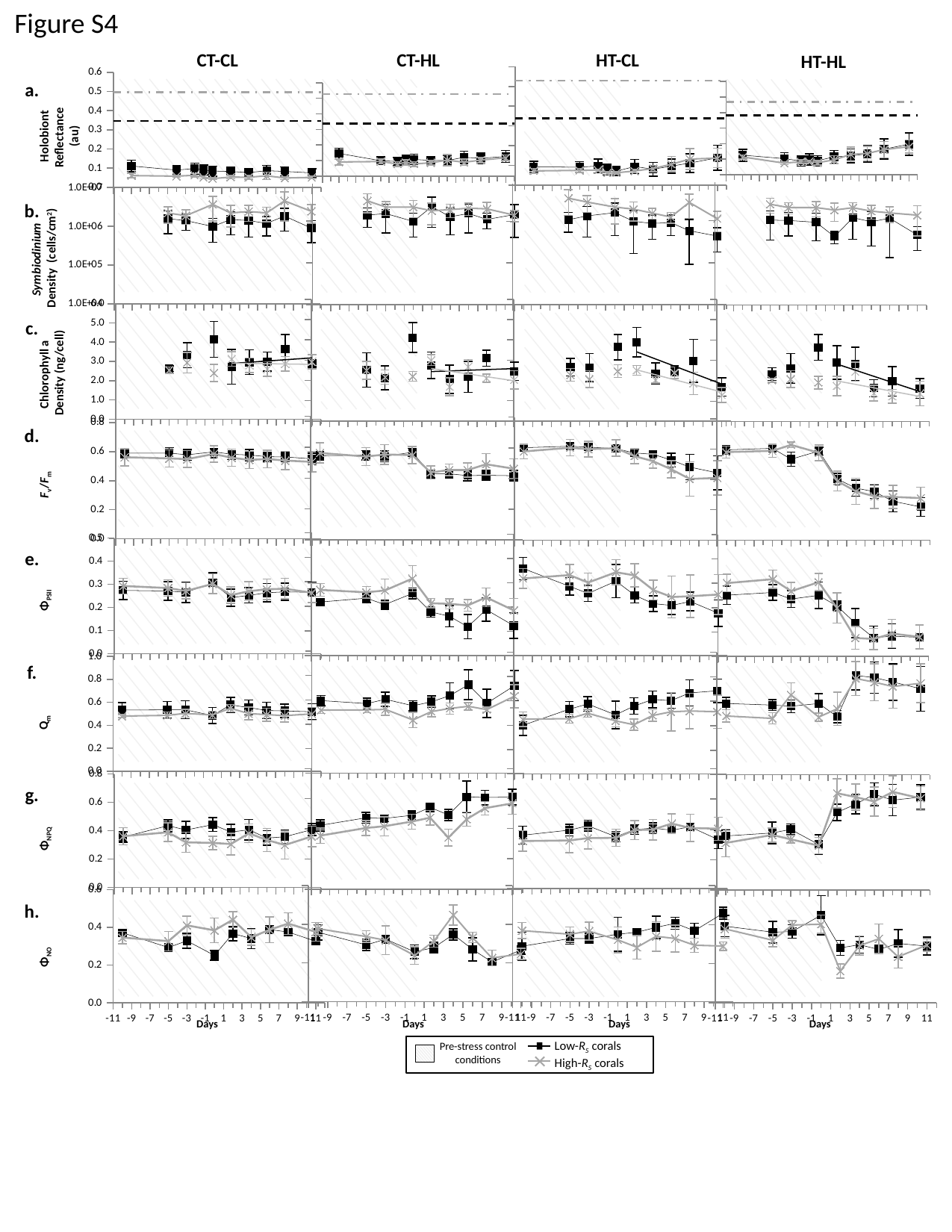

Figure S4
CT-CL
CT-HL
HT-CL
HT-HL
### Chart
| Category | high Rs | low Rs | High | Low |
|---|---|---|---|---|
### Chart
| Category | high Rs | low Rs | High | Low |
|---|---|---|---|---|
### Chart
| Category | high Rs | low Rs | High | Low |
|---|---|---|---|---|
### Chart
| Category | high Rs | low Rs | High | Low |
|---|---|---|---|---|a.
Holobiont Reflectance
(au)
### Chart
| Category | high Rs | low Rs |
|---|---|---|
### Chart
| Category | high Rs | low Rs |
|---|---|---|
### Chart
| Category | high Rs | low Rs |
|---|---|---|
### Chart
| Category | high Rs | low Rs |
|---|---|---|Symbiodinium
Density (cells/cm2)
b.
### Chart
| Category | high Rs | low Rs | high before stress | low below stress |
|---|---|---|---|---|
### Chart
| Category | high Rs | low Rs | high before stress | low below stress |
|---|---|---|---|---|
### Chart
| Category | high Rs | low Rs | high before stress | low below stress |
|---|---|---|---|---|
### Chart
| Category | high Rs | low Rs | high before stress | low below stress |
|---|---|---|---|---|c.
Chlorophyll a
 Density (ng/cell)
### Chart
| Category | high Rs | low Rs |
|---|---|---|
### Chart
| Category | high Rs | low Rs |
|---|---|---|
### Chart
| Category | high Rs | low Rs |
|---|---|---|
### Chart
| Category | high Rs | low Rs |
|---|---|---|d.
Fv/Fm
### Chart
| Category | high Rs | low Rs |
|---|---|---|
### Chart
| Category | high Rs | low Rs |
|---|---|---|
### Chart
| Category | high Rs | low Rs |
|---|---|---|
### Chart
| Category | high Rs | low Rs |
|---|---|---|e.
ΦPSII
### Chart
| Category | high Rs | low Rs |
|---|---|---|
### Chart
| Category | high Rs | low Rs |
|---|---|---|
### Chart
| Category | high Rs | low Rs |
|---|---|---|
### Chart
| Category | high Rs | low Rs |
|---|---|---|f.
Qm
### Chart
| Category | high Rs | low Rs |
|---|---|---|
### Chart
| Category | high Rs | low Rs |
|---|---|---|
### Chart
| Category | high Rs | low Rs |
|---|---|---|
### Chart
| Category | high Rs | low Rs |
|---|---|---|g.
ΦNPQ
### Chart
| Category | high Rs | low Rs |
|---|---|---|
### Chart
| Category | high Rs | low Rs |
|---|---|---|
### Chart
| Category | high Rs | low Rs |
|---|---|---|
### Chart
| Category | high Rs | low Rs |
|---|---|---|h.
ΦNO
Days
Days
Days
Days
Low-RS corals
High-RS corals
Pre-stress control conditions
×
