# Supplementary material for: Skeletal light-scattering accelerates bleaching response in reef-building corals
Source: BMC Ecol. 2016 Mar 21;16:10. doi: 10.1186/s12898-016-0061-4 (PMC4800776; doi:10.1186/s12898-016-0061-4)
Supplement: Supplementary file 6 — 10.1186/s12898-016-0061-4 Supporting Text. Supporting methods (1) and supporting references (2). [file 12898_2016_61_MOESM6_ESM.docx]

**Supporting Text**

**Skeletal light-scattering accelerates bleaching response in reef-building corals**

Timothy D. Swain^1,2^, Emily DuBois^1,2^, Andrew Gomes^3^, Valentina P. Stoyneva^3^, Andrew J. Radosevich^3^, Jillian Henss^1,2^, Michelle E. Wagner^1,2^, Justin Derbas^3^, Hannah W. Grooms^1^, Elizabeth M. Velazquez^1^, Joshua Traub^1^, Brian J. Kennedy^1^, Arabela A. Grigorescu^4^, Mark W. Westneat^2^, Kevin Sanborn^5^, Shoshana Levine^5^, Mark Schick^5^, George Parsons^5^, Brendan C. Biggs^6^, Jeremy D. Rogers^3^, Vadim Backman^3^, Luisa A. Marcelino^1,2^

**^1^**Department of Civil and Environmental Engineering, Northwestern University, 2145 Sheridan Road, Evanston, Illinois, 60208, United States of America, **^2^**Department of Zoology, Field Museum of Natural History, 1400 South Lake Shore Drive, Chicago, Illinois, 60605, United States of America, **^3^**Department of Biomedical Engineering, Northwestern University, 2145 Sheridan Road, Evanston, Illinois, 60208, United States of America, **^4^**Keck Biophysics Facility, Northwestern University, 633 Clark Street, Evanston, Illinois, 60208, United States of America,**^5^**Fishes Department, John G. Shedd Aquarium, 1200 South Lake Shore Drive, Chicago, Illinois, 60605, United States of America,**^6^**Division of Water Resource Management, Florida Department of Environmental Protection, 2600 Blair Stone Road, Tallahassee, 32399, United States of America.

**1. Supporting methods**

**1.1.** **Holobiont Reflectance, *R_H_*, and Skeletal Reflectance, *R_S_*.** Holobiont reflectance of live corals mounted to tiles was measured *in situ* throughout the experiment (Additional file 9: Figure S6e), identically to the collection of skeletal reflectance. In the visible region, *Symbiodinium* photopigments are the main absorbers (chlorophyll *a*, with absorption maximum peaks at 435–440nm and 675nm [Additional file 1: Figure S1], peridinin at 470–490 nm, and chlorophyll *c_2_* at 450–460 and 630nm [19, 83, 84]), while some host pigments and endolithic algae can also absorb in the visible [19, 83]. As corals bleached and less than 10% of symbionts remained associated with the host, *R_H_* approached the value for its respective *R_S_* (Figure 1e, Additional file 1: Figure S1). However, in the NIR region, *R_H_* sometimes exceeded *R_S_*, especially in bleached corals (Additional file 1: Figure S1) which is in agreement with previously observed spectra of coral species at different depths and geographic areas [28, 85-89]. Due to a combination of factors, the end result may be a situation where the coral tissue is more reflective than the skeleton (*R_H_* > *R_S_*). Within tissue scalar irradiances across visible and NIR regions may differ across and within corals species due to differences in skeletal reflectance and *µ_S_ʹ* [16, 30, 83], presence of coral pigments [33, 85, 87, 89] which can be highly scattering and reflective [33] and light scattered in the tissue leading to lateral redistribution within the tissue [19, 28]. These spectra are quantitatively and qualitatively consistent with previously reported values for healthy and bleached corals [15, 29, 38, 85] where the region of lowest reflectance corresponds to the region of maximum absorption of chlorophyll *a* (675nm) in healthy corals (Additional file 1: Figure S1c, e).

Skeletal reflectance, *R_S_*, was measured in coral skeletons cleaned with pressurized artificial seawater, soaked for < 12 h in 3% sodium hypochlorite, rinsed, and dried. When a skeleton is modeled as a semi-infinite randomly homogeneous turbid medium (i.e. without structures that function as optical fibers that non-randomly channel light), *R_S_* is related with *µ_S_ʹ* and the absorption coefficient *µ_a_* as follows: $R_{s}=e^{-\frac{A}{\sqrt{1+\frac{{\mu'}_{s}}{\mu_{a}}}}}$ , where empirical constant *A* ~ 4.5 [90]. For a typical skeleton, *µ_a_* is much smaller than *µ_S_ʹ* (*µ_a_* < 0.01*µ_S_ʹ*) and is not a significant factor in short-path light transport (measured by *µ_S_ʹ_,m_*) but may substantially reduce *R_S_* [18]. For non-flat skeletal morphologies, *R_S_* is determined by *µ_S_ʹ*, *µ_a_* and coral morphology (colony growth form and microstructures) [27, 29-31, 91].

**1.2. DNA extraction, amplification, sequencing, and identification.** Nucleic acids were extracted using a modified cetyltrimethyl-ammonium bromide (CTAB) technique [60]. *Symbiodinium* nuclear internal transcribed spacer (ITS) region 2 and chloroplast 23S ribosomal DNA (rDNA), as well as Scleractinia mitochondrial cytochrome oxidase I (COI), cytochrome b (CytB), and nuclear ITS were targeted because they are commonly used to identify and address evolutionary questions within these taxa [79-82]. Markers were selectively amplified by polymerase chain reaction (PCR) using standard reagents (Invitrogen) and the primers and annealing temperatures listed in Table S1a. PCR products were separated by gel electrophoresis and directly sequenced in the forward and reverse directions using the amplification primers and Big-Dye^®^ Terminator (Applied Biosystems) chemistry. Sequences were assembled and edited using SEQUENCHER 4.0.5 (Gene Codes Co.), and aligned using BioEdit 7.0.5.2. ITS sequences identified from single bands were assumed to represent dominant *Symbiodinium* phylotypes. Comparisons between experimental *Symbiodinium* nucleotide sequences and Genbank accessions identified clades and phylotypes with > 95% sequence similarity (Table 1). Comparisons between experimental coral nucleotide sequences and Genbank accessions corroborated morphological identifications with > 95% sequence identity match (with the exception of *S. hystrix* with 87% ITS identity match) to target species represented in Genbank (Table 1).

**1.3. Aquarium conditions.** The two experimental aquaria are 1,020 L (each kept at 420 L, or ~ 25 cm depth, to increase flow velocity) recirculating (through an aquarium protein skimmer and 340 L reservoir) baffled flumes with uniform unidirectional flow (2.5 - 4 cm/s) of filtered artificial seawater (Additional file 9: Figure S6a, d). Reservoir and aquaria temperatures were constantly measured (± 1°C) with a digital thermometer (Oregon Scientific, model THT312). Aquaria were monitored and adjusted daily for salinity and weekly for pH, phosphates, nitrates, nitrites, ammonium, calcium, and alkalinity; with weekly partial siphoning and a 50% water change each month leading up to the experiment. Corals were daily fed live naupli and rotifer cultures prior to the experiment, but were not fed during the experiment.

Corals mounted to tiles rested on plastic grids 11 cm from the bottom of the aquaria under a divided (by suspended shade of black aluminum foil) array of lamps that allowed independent control of light conditions in two different sectors of each aquarium (Additional file 9: Figure S6a). The arrays contained two 400 W, 10,000 K bulbs (Aqualite™ metal halide, USHIO America, Inc.) in the control sector and one 1000 W, 10,000 K bulb (SunMaster™ metal halide, Venture Lighting International, Inc.) in the high-light sector; each array was controlled by a 400–1000 watt electronic ballast (Galaxy Select-A-Watt Turbo Charge™, Sunlight Supply, Inc.). The 10,000K lamp bulbs have a high color temperature which simulates sunlight near the equator at approximately 5 meters depth. Light intensity maps of each sector (experimental condition) were created using a IL420A Radiometer, International Light Technologies Inc., at 17 cm from the bottom of the aquaria with 48 measurements per sector, spaced every 10 cm^2^. Light intensity maps were generated at three different times of the experiment; prior, during, and after the initiation of stress. Before initiation of thermal and light stress, all sectors (high-light arrays were shaded to mimic control-light conditions) were illuminated at 83.1 ± 1 µmol photons m^2^/s for 10 days and pre-experimental (i.e. baseline) measurements were collected for all response variables (Additional file 2: Figure S2).

Explants were assigned to light sectors in both aquaria so that they would contain 8 ramets of each coral species to provide replicates for destructive sampling throughout the experiment and were randomly distributed within a sector to acclimate for about three weeks (Additional file 9: Figure S6d). Baseline physiological measurements for the 10 coral species studied were collected for all response variables starting at 6–10 days before the experiment. The randomly assigned position of each explant in the tank was kept constant throughout the entire experiment and recorded so that after the experiment ended the cleaned skeletons could be taken back to the same position of their respective explants for *R_S_* measurement.

**1.4. Pulse-amplitude modulation chlorophyll fluorometry.** *Symbiodinium* photosynthetic efficiency was assessed through pulse-amplitude modulation (PAM) chlorophyll fluorometry (Junior PAM; Walz, Germany). The optical fiber was immobilized in a black polyvinyl chloride (PVC) tube held at a 23° angle by a machined acrylic block (custom-built for this study) that slid over a square PVC post attached to the coral-mounting tile and rested on a PVC pipe sleeve (design and angle of measurement following R. Iglesias-Prieto, personal communication; Additional file 9: Figure S6b, c). The PAM probe must be held at a non-vertical angle to avoid artificially shading targeted tissues (with the probe or its holder and mount) during induction curve analysis, however the angle of both the probe and mounted coral tissues ensured that measurements were collected from illuminated regions of the colony. The probe holder stably fixed the PAM optical fiber 1–3 mm from the coral surface during measurements and allowed the probe to be returned to each explant with the fiber-tip in the same three-dimensional geometry as previous measurements; reducing noise and increasing stability of measurements (R. Iglesias-Prieto, personal communication). This design favors standardization and reproducibility over the ability to differentiate between tissue types or regions of the colony, both of which would have required an impractical amount of measurements given the number of species and conditions examined.

**1.5 Maximum excitation pressure over photosystem II, *Q_m_*.**

*Symbiodinium* exhibit $\Phi_{PSII}$ oscillations as a result of the induction of multiple photoprotective pathways that compete for energy dissipation when light absorption exceeds photochemistry [40]. This can be measured as maximum excitation pressure over photosystem II, *Q_m_*, (1 – [($\Phi_{PSII}$ *_at peak light_*)/(*F_v_/F_m_* *_at dawn_*)]) [40, 41]; an alternative to the original conceptualization [92] that required constant optical geometry over a 24 h period [93]. Values of *Q_m_* are correlative, but not equivalent [40], to the non-photochemical quenching coefficient (total of non-photochemical mechanisms that quench singlet-excited chlorophylls) and are useful as an indication of photo-physiological performance that distinguishes between light-limitation (*Q_m_ ≈* 0, most reaction centers remain open), photoacclimation (*Q_m_* remains unchanged during suppressed photochemical efficiency), and photoinhibition (*Q_m_* ≈ 1, most reaction centers are closed) by indicating the proportion of open PSII reaction centers under maximal irradiance [11, 12, 40].

**1.6. *Symbiodinium* density.** Colonies were destructively sampled by transferring individual explants from tiles to 400 ml polypropylene cups filled with aquarium water for < 1 h before processing. Coral and *Symbiodinium* cells were removed from the skeleton by directional high-pressure artificial seawater and the resulting tissue slurry was concentrated to 2 ml by centrifugation (500 x G for 5 min). The concentrated extract was divided into 2 aliquots: 0.5 ml was stored in 1.5 ml polypropylene microcentrifuge tubes for < 24 h at 4°C before *Symbiodinium* cell counts were completed, and 1.5 ml was pelleted by centrifugation and stored for < 3 days at -80°C for high-performance liquid chromatography (HPLC) analysis of photosynthetic pigment identities and concentrations. Denuded skeletons were soaked for < 12 h in a 3% sodium hypochlorite solution, rinsed with freshwater, and dried for surface area estimation using a single-dip wax method [69].

After dilution (or concentration) to achieve an average of 150 cells per field, six replicates of isolated *Symbiodinium* were resuspended in sea water, loaded into a hemocytometer, and digitally photographed through a compound microscope. Digital images of cells were enumerated using the cell count algorithms of ImageJ (version 1.47; NIH) and were converted to densities by normalizing total cell counts (corrected for dilution) to total surface area of each ramet.

**1.7. Photosynthetic pigment concentration.** Corals were processed for photosynthetic pigment concentration analysis as described in Text S1.6. Photosynthetic pigments were extracted and chromatographically separated using established procedures and gradients [68] with modifications: a liquid chromatograph (Hewlett-Packard 1100 series) with diode-array detector and thermostated autosampler was used with a thermostated 3 x 250 mm reverse phase column with 5µm particles (Waters Symmetry C18) and a 4.6 x 10 mm guard column (Waters Spherisorb ODS2). Photosynthetic pigments were detected at the following wavelengths: chlorophyll *c_2_* (450 nm), peridinin (472 nm), chlorophyll *a* (665nm), pheophytin *a* (665 nm), diadinoxanthin (450 nm) and diatoxanthin (450 nm).

HPLC calibration curves were constructed using serial dilutions of chlorophyll *a*, chlorophyll *c_2_*, pheophytin *a*, peridinin, diadinoxanthin, and diatoxanthin, pigment standards (DHI Water and Environment). The range of peak absorbances over which the calibration regression equation would be valid was determined using serial dilutions of extracted phytopigments from fully pigmented (*Acropora muricata*) and bleached (*Stylophora pistillata*) corals with triplicate HPLC runs of independent dilutions. We obtained a linear relationship between the concentration of pigments and the peak area over a range of 120 fold (100 to 12,000 arbitrary units), which was well within the physiological range of healthy and bleached specimens (> 90% loss in *Symbiodinium* density as determined by cell counts, see Text S1.6). Isolated *Symbiodinium* were transferred to liquid nitrogen storage (-196°C) for less than 3 months prior to extraction, identification, and quantification of photopigments. *Symbiodinium* pellets were transferred to 5 ml glass vials and extracted using 2 ml of HPLC-grade methanol (Sigma-Aldrich) under low light (to minimize degradation of photopigments). After the addition of methanol, the extraction was agitated for 120 seconds every 30 minutes for 2 hours, before adding 220 μl of 0.5 ammonium acetate (Sigma-Aldrich) and agitating again; refrigerating the vials in the dark after every agitation. Residual cell material was removed from the post-extract (300 µl) through centrifugation at 1000 x G for 3 minutes, and the supernatant (100 µl) was transferred to amber HPLC vials for injection (50 µl).

**1.8. Statistical analysis.** The effects of potential bleaching explanatory variables (*µ_S_ʹ_,m_* , *R_S_*_,_ and *Symbiodinum* thermotolerance) and response variables (*F_v_/F_m_*, and *Q_m_* ) were individually evaluated using a linear mixed model (LMM) in Stata 11.2. The effects of time, light, and temperature stress were also analyzed by LMM. In brief, LMM is an extension of linear regression and it accounts for the hierarchical organization of data [70]; longitudinal measurements taken on individual coral ramets which were nested within different coral species. The potential explanatory variables listed above were considered fixed effects while random effects for both coral ramets and coral species were used in the LMM to account for correlated errors within repeated measurements on the same ramet and measurements on the same species. Both an unstructured covariance matrix for the random effects associated with species level and maximum restricted likelihood estimation were used. To reduce potential confounding by non-linear behavior of the *F_v_/F_m_* and *Q_m_* versus time curve, the time variable was restricted to days 0–6 after the application of stress conditions to capture only the linear portion of the *F_v_/F_m_* and *Q_m_* response. An interaction term for time and *µ_S_ʹ_,m_* was included in the model to capture the apparent difference in the temporal slope for corals with high- and low-scattering skeletons. If a significant interaction was found, group differences over time were further analyzed by marginal analysis. While *Q_m_* values were analyzed by marginal analysis as raw values, *F_v_/F_m_* values were normalized to initial values; although low-*µ_S_ʹ_,m_* coral have higher *F_v_/F_m_* to start, with the application of thermal- and light-stress they cross the *F_v_/F_m_* curve for high-*µ_S_ʹ_,m_* corals at day 4 (Figure 1b, Additional file 2: Figure S2), making marginal analysis insensitive to absolute differences over time.

**1.9. *Skeleton-dependent light absorption model***

We developed a novel model of *Symbiodinium* light absorption, which accounts for skeleton-driven absorption and multiple reentry effects that have not been previously modeled. Incident light absorption by *Symbiodinium* (fraction $I_{a}$) can be viewed as the result of skeleton-independent absorption (fraction $I_{a1}$) of downwelling light and skeleton-dependent absorption (fraction $I_{a2}=I_{a}-I_{a1}$) of light reflected by the skeleton [15-17]. Light that is not absorbed in the first pass (fraction $1-I_{a1}$of the incident light) can be reflected by the skeleton back into the tissue by multiple light scattering, lost to skeletal absorption, or diffusely scattered out of the colony [17, 19, 27, 28]. This process may continue due to multiple reentries of unabsorbed light back into the skeleton (i.e. aided by coral morphology) [19, 94]. Thus, skeleton-dependent absorption might be due to single or multiple passes of light through tissue due to multiple reflections by the skeleton [15, 17]. Because direct quantification of light absorption by pigments in live corals is not currently possible, we developed an empirical model relating $I_{a1}$ and $I_{a2}$ with parameters that were experimentally measured: skeletal reflectance (*R_S_*) and holobiont reflectance (*R_H_*) (measured at different time points throughout the experiment, *R_H_(t)*).

Starting with a balance equation:

$R_{H}=R_{1}\left( 1-I_{a1} \right)\left( 1-a_{2} \right)$, (4)

where *R_1_* is the fraction of unabsorbed light that is leaving the holobiont after being reflected by the skeleton back into tissue including all reentries and $a_{2}$ is the fraction of this reflected light that is absorbed by the pigments in the tissue. In a special case where multiple reentry is not feasible (flat coral model), ${R_{1}=R}_{S}$. This is a non-linear equation for $I_{a1}$ since $a_{2}$ depends on $I_{a1}$. Introducing new notations $R'={R_{H}/R}_{S}$, $\beta={R_{S}/R}_{1}$ and $\alpha={a_{2}/I}_{a1}$ and solving equation (4) for $I_{a1}$, we get:

$I_{a1}=\frac{1}{2\alpha}\left( 1+\alpha-\sqrt{\left( 1+\alpha\right)^{2}-4\alpha\left( 1-\beta R' \right)} \right)$ (5)

In order to find the total absorbed intensity (and thus Ia_2_), we write another balance equation:

$I_{a}=I_{a1}+\left( 1-I_{a1} \right)R_{1}a_{2}+\left( 1-I_{a1} \right)\gamma\left( R_{S}-R_{1} \right)$. (6)

Here the first term ($I_{a1}$) is the absorption of downwelling light, second term is the absorption of light leaving the holobiont after being reflected by the skeleton, and the third term describes light absorbed due to multiple reentry. The term $\gamma\left( R_{S}-R_{1} \right)$ describes the fraction of light that is absorbed by tissue through processes other than $I_{a1}$ or $a_{2}$ through coefficient *γ*. The third term vanishes for a flat coral model. Rearranging equation (6), we find the skeleton-dependent absorption:

$I_{a2}=I_{a}-I_{a1}=\left( 1-I_{a1} \right)\left( \frac{\alpha}{\beta}I_{a1}+\gamma\frac{\beta-1}{\beta} \right)R_{S}$, (7)

If $R'$ and *R_S_* are known, $I_{a1}$ and $I_{a2}$ can be found using equations (5) and (7), respectively. Coefficients *α*, *β*, and *γ* depend on the geometry of the coral, the optical properties of the skeleton, and the concentration of absorbing pigments in tissue; thus, they also depend on time *t*.

Coefficient *α* describes the amplification of light absorption due to elongation of light paths through the tissue caused by diffuse skeletal reflection of unabsorbed downwelling light. Alpha must be greater than unity; *α* of 1 is only feasible if the skeleton reflects light as a mirror without redirection [15]. In the special case of a flat coral model, $1<\alpha<2$ and *α* increases as the concentration of absorbing pigments is decreased. This can be illustrated using the flat coral model [15, 17, 18]; light reflected orthogonally to the surface of the coral has the shortest path through the tissue and lowest probability of absorption (compared to light reflected at greater angles, which have elongated paths). The smaller the optical thickness of the tissue ($\tau=L\mu_{a}$ with thickness *L* and absorption coefficient *μ_a_*), the greater the difference in absorption due to path length difference [17, 18]. The value of $\alpha=2$ corresponds to the limit of $\tau0$ in a flat coral model with Lambertian reflection. Even longer paths can be created by non-flat skeletons due to multiple reentry of light and $\alpha>2$ [15] ($\alpha\gg1$ is artificially created in a laser cavity). In the limit of low pigment concentration ($\tau0$), light absorption amplification (ratio of the light absorbed in the presence of skeleton to that without) is ${A=1+\alpha R}_{S}$. Coefficients *β* and *γ* are related to the non-flatness of the skeleton and account for the reentry effect; $\beta=\gamma=1$ for flat corals, $\beta>1$ and $\gamma<1$ for non-flat geometries.

If reentry is neglected ($\beta=\gamma=1$), the total absorption (Eqs. 5–7) converges to a simpler solution that has been previously introduced and conventionally used to estimate the absorption of light by tissue pigments based on the measurements of holobiont and skeletal reflectance values [15, 29, 49], $I_{a}\approx1-R'$ , if one of the following two conditions is satisfied: $I_{a2}$ can be neglected (e.g. $\tau\gg1$ and most of the absorption is due to the downwelling light) or $R_{S}=1$. This simplified equation (${I_{a}}_{simplified}\equiv1-R'$ ) overestimates $I_{a}$ given by Eqs. 5-7 by, approximately $I_{a2}$, with the discrepancy most pronounced for lower *R_S_*: $\frac{I_{a_{simplified}}}{I_{a}}-1\sim\frac{1-R_{S}}{1+R_{S}}$ for $\tau\ll1$. Due to this discrepancy, and because our goal was to estimate the effect of skeletal-dependent absorption, our analysis uses the full equations (5–7).

Even though the coefficients *α*, *β*, and *γ* depend on the optical properties of the skeletons and the concentration of pigments, the model can be used to estimate the range of $I_{a1}$ and $I_{a2}$. Indeed, the skeleton-dependent absorption increases with *α* (e.g. as symbionts leave). Thus, we can obtain the conservative (lower) bound on $I_{a2}$ by taking $\alpha=1$. The upper bound on $I_{a2}$ can be obtained for $\alpha=2$. We estimated the lower bound on the skeleton-dependent absorption by using equations (5) and (7) with $\alpha=\beta=\gamma=1$.

**1.10. Analysis of taxon-specific bleaching response index (BRI) as a predictor of experimental bleaching.**

We also considered a taxon-specific bleaching response index (BRI) as a predictor of experimental bleaching. The BRI was constructed for 94 coral taxa with 1,412 unique records of taxon-specific bleaching severity and related mortality from mass bleaching events throughout the tropics from 1982–2005 [18]. As expected, BRI correlated with the bleaching response (quantified by *ΔPE*, r^2^ = 0.23; data not shown) and higher BRI values corresponded to more severe bleaching (lower negative *ΔPE*), but failed to reach statistical significance (*p* = 0.22) for the difference between mean *ΔPE* of low- and high-BRI groups. Correlation between BRI and experimental bleaching response is expected from the correlation study of µ_S_ʹ_,m_ and BRI, that identified µ_S_ʹ_,m_ as a reliable predictor of BRI [18]. This study also corroborated these earlier results: µ_S_ʹ values of the 10 corals could predict BRI (r^2^ = 0.58), where lower *µ_S_ʹ_,m_* is associated with higher BRI. However, BRI is not an intrinsic property of corals, but rather a metric of mean bleaching severity of its taxon (species- or genus-level); therefore the bleaching severity of the experimental corals was best predicted by *µ_S_ʹ_,m_* (as an intrinsic property of individual colonies).

**2. Supporting References**

83. Magnusson SH, Fine M, Kühl M (2007) Light microclimate of endolithic phototrophs in the scleractinian corals *Montipora monasteriata* and *Porites cylindrica*. Mar Ecol Prog Ser 332:119–28.

84. Venn AA, Wilson MA, Trapido-Rosenthal HG, Keely BJ, Douglas AE (2006) The impact of coral bleaching on the pigment profile of the symbiotic alga, *Symbiodinium*. Plant Cell Environ 29:2133–42.

85. Hochberg EJ, Atkinson MJ, Apprill A, Andréfouët S (2004) Spectral reflectance of coral. Coral Reefs 23:84–95.

86. Kahng SE, Hochberg EJ, Apprill A, Wagner D, Luck DG, Perez D, Bidigare RR (2012) Efficient light harvesting in deep-water zooxanthellate corals. Mar Ecol Prog Ser 455:65–77.

87. Mazel CH, Fuchs E (2003) Contribution of fluorescence to the spectral signature and perceived color of corals. Limnol Oceanogr 48:390–401.

88. Myers MR, Hardy JT, Mazel CH, Dustan P (1999) Optical spectra and pigmentation of Caribbean reef corals and macroalgae. Coral reefs 18:179–86.

89. Stambler N, Shashar N (2007) Variation in spectral reflectance of the hermatypic corals, *Stylophora pistillata* and *Pocillopora damicornis*. J Exp Mar Biol Ecol 2007;351:143–149.

90. Madsen S, Wilson B, Patterson M, Park Y, Jacques S, Hefetz Y (1992) Experimental tests of a simple diffusion-model for the estimation of scattering and absorption-coefficients of turbid media from time-resolved diffuse reflectance measurements. Appl Optics 31:3509–17.

91. Anthony KRN (1999) A tank system for studying benthic aquatic organisms at predictable levels of turbidity and sedimentation: Case study examining coral growth. Limnol Oceanogr 44:1415–22.

92. Maxwell DP, Falk S, Huner NPA (1995) Photosystem-II excitation pressure and development of resistance to photoinhibition. 1. Light-harvesting complex-II abundance and zeaxanthin content in *Chlorella vulgaris*. Plant Physiol 107:687–94.

93. Gorbunov MY, Kolber ZS, Lesser MP, Falkowski PG (2001) Photosynthesis and photoprotection in symbiotic corals. Limnol Oceanogr 2001;46:75–85.

94. Kaniewska P, Anthony KRN, Hoegh-Guldberg O (2008) Variation in colony geometry modulates internal light levels in branching corals, *Acropora humilis* and *Stylophora pistillata*. Mar Biol 155:649–60.
