# Supplementary figures and images for: Skeletal light-scattering accelerates bleaching response in reef-building corals
Source: BMC Ecol. 2016 Mar 21;16:10. doi: 10.1186/s12898-016-0061-4 (PMC4800776; doi:10.1186/s12898-016-0061-4)

## Slide 1
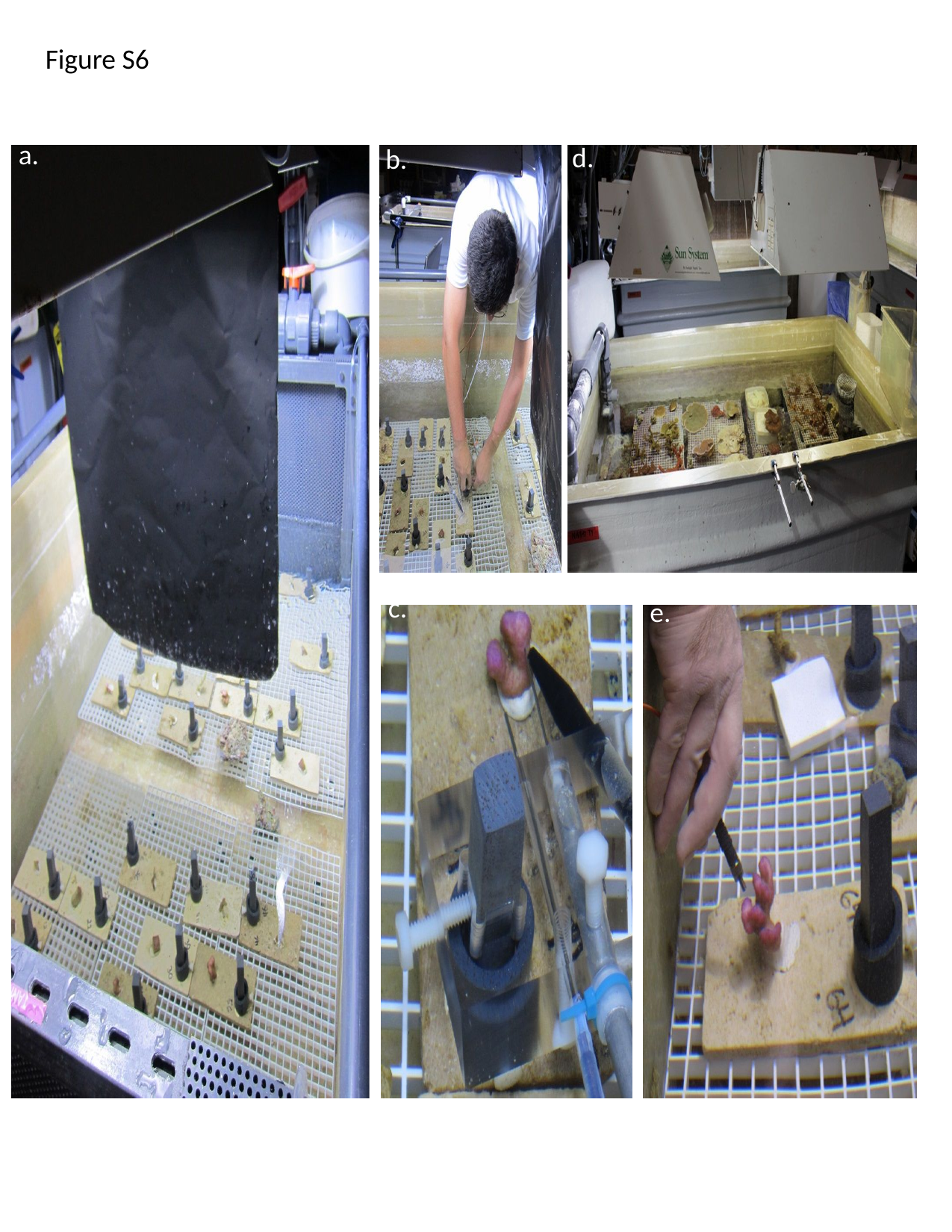

Figure S6
a.
d.
b.
d.
c.
e.

Supplement: Supplementary file 9 — 10.1186/s12898-016-0061-4 Experimental setup. High temperature aquarium encompassing the HT-CL and HT-HL conditions on day 11 of the bleaching experiment (a); black divider separating light arrays, flow baffles, and mounted corals can be seen. Collecting PAM measurements in the control temperature aquarium within the CT-CL condition (CT-HL on the opposite side of black divider) on day 11 of the bleaching experiment (b); positioning of the PAM instrument probes above the coral explants mounted on stone tiles can be seen. Close up of probe holder (custom-machined acrylic block that ensures probes are returned to each explant in the same three-dimensional geometry as previous measurements) supported at a 23° angle by square PVC post (gray) attached to the coral-mounting tile (c); probes are (left to right) temperature, O2 (data not reported), and PAM fiber optic immobilized in a black PVC tube. Control temperature aquarium encompassing the CT-CL and CT-HL conditions (HT aquarium in background) during acclimation period prior to prescreening and fragmentation (d); photograph taken before installation of the flow baffles and black divider separating light arrays. Hand-held optical fiber attached to a spectrometer to measure R H (and R S of cleaned skeleton) with white reflectance standard visible in the background (e). Consent to publish these images has been documented. [file 12898_2016_61_MOESM9_ESM.pptx]
